# Spectrofluorimetric and Computational Investigation of New Phthalimide Derivatives towards Human Neutrophil Elastase Inhibition and Antiproliferative Activity

**DOI:** 10.3390/ijms24010110

**Published:** 2022-12-21

**Authors:** Beata Donarska, Marta Świtalska, Joanna Wietrzyk, Wojciech Płaziński, Krzysztof Z. Łączkowski

**Affiliations:** 1 Department of Chemical Technology and Pharmaceuticals, Faculty of Pharmacy, Collegium Medicum, Nicolaus Copernicus University, Jurasza 2, 85-089 Bydgoszcz, Poland; 2 Hirszfeld Institute of Immunology and Experimental Therapy, Polish Academy of Sciences, Rudolfa Weigla 12, 53-114 Wrocław, Poland; 3 Jerzy Haber Institute of Catalysis and Surface Chemistry, Polish Academy of Sciences, Niezapominajek 8, 30-239 Cracow, Poland; 4 Department of Biopharmacy, Medical University of Lublin, Chodzki 4a, 20-093 Lublin, Poland

**Keywords:** spectrofluorimetric method, human neutrophil elastase, antiproliferative activity, phthalimide, thiazole

## Abstract

Herein, nine phthalimide-based thiazoles (**4a**–**4i**) were synthesized and investigated as new human neutrophil elastase (HNE) inhibitors using spectrofluorimetric and computational methods. The most active compounds containing 4-trifluoromethyl (**4c**), 4-naphthyl (**4e**) and 2,4,6-trichloro (**4h**) substituents in the phenyl ring exhibited high HNE inhibitory activity with IC_50_ values of 12.98–16.62 µM. Additionally, compound **4c** exhibited mixed mechanism of action. Computational investigation provided a consistent picture of the ligand-receptor pattern of inter-actions, common for the whole considered group of compounds. Moreover, compounds **4b**, **4c**, **4d** and **4f** showed high antiproliferative activity against human cancer cells lines MV4-11, and A549 with IC_50_ values of 8.21 to 25.57 µM. Additionally, compound **4g** showed high activity against MDA-MB-231 and UMUC-3 with IC_50_ values of 9.66 and 19.81 µM, respectively. Spectrophotometric analysis showed that the most active compound 4c demonstrated high stability under physiological conditions.

## 1. Introduction

Tumor cells interact with surrounding tissues leading to changes in their structure and biochemistry, resulting in a specific tumor microenvironment that can stimulate tumor growth. The tumor microenvironment includes the surrounding blood vessels, immune cells, fibroblasts, signaling molecules and the extracellular matrix (ECM). Recently, processes in the tumor microenvironment have been an important point of interest in the search for new anticancer drugs [1]. In this context, neutrophils, which in response to cytokines and chemokines secreted by the tumor are released from the bone marrow and richly infiltrate the tumor microenvironment, become interesting. Increased activity of tumor-associated neutrophils (TANs) has been shown to be associated with faster tumor progression, angiogenesis and reduced survival in various types of cancer [2]. 

Tumor-associated neutrophils are capable of forming neutrophil extracellular traps (NETs), which under physiological conditions play an important role in defense against infection, and in the case of tumors can stimulate metastasis and angiogenesis [3]. NETs consist of secreted nuclear DNA supplied with proteases and various inflammatory mediators [4]. 

An important component of NETs are neutrophil serine proteases (NSPs), which include neutrophil elastase (NE), cathepsin G and proteinase 3. These homologous enzymes belong to the chymotrypsin family, which contains aspartate, histidine and serine residues in the active site [5]. The most abundant serine protease in neutrophils is elastase, which has broad substrate specificity [6]. Potential substrates of NE are components of the extracellular matrix mainly elastin, fibrin, fibronectin, as well as various proteins, i.e., coagulation factors, immunoglobulins and cytokines. Under physiological conditions, elastase together with another neutrophil serine proteases, plays an important role in the immune response during infection or in modulating the inflammatory process, and its activity is controlled by its endogenous inhibitors, i.e., α1-antitrypsin or elafin [7,8]. An imbalance between elastase activity and its inhibitors can cause many inflammatory pathological processes such as cystic fibrosis, chronic obstructive pulmonary disease, atherosclerosis or arthritis [9,10,11]. Increased elastase activity has also been marked in the pathomechanism of various types of cancer. Using the LSK-K-ras mouse model of lung adenocarcinoma as an example, it was shown that mice lacking the ELANE gene (encoding neutrophil elastase) had a higher surface survival rate compared to ELANE (+) mice. In addition, NE was shown to stimulate tumor cell proliferation by degrading the insulin receptor substrate-1 (IRS-1), resulting in an increased interaction between phosphatidylinositol kinase-3 (PI3K) and platelet-derived growth factor receptor (PDGFR) [12]. Neutrophil elastase also plays an important role in the development of acute promyelocytic leukemia (APL). It has been recently shown that neutrophil elastase stimulates proliferation and exhibits anti-apoptotic effects in cells of leukemia lines K562 and U937, and that the treatment of cells with the specific elastase inhibitor GW311616A led to inhibition of cell growth and induction of apoptosis [13]. Another study demonstrated that increased NE expression and levels were also found in tissues and sera collected from patients with colorectal cancer (CRC). In addition, tumor volume was compared in two xenografts with and without Sivelestat administration. The results proved that Sivelestat, an elastase inhibitor, significantly reduced tumor growth [14]. In breast cancer, increased elastase activity was found in tumor cells and overexpression of elafin, a specific elastase inhibitor, resulted in inhibition of proliferation and reduction in tumor size [15]. It has also been confirmed that neutrophil elastase stimulates proliferation, migration and invasion of other types of cancer cells including prostate cancer and pancreatic ductal adenocarcinoma (PDAC) [16,17].

Taking into account the involvement of elastase in the development of many types of cancer, it becomes interesting to search for new compounds with anticancer activity with synergistic elastase inhibitory activity. Among the already known elastase inhibitors are such compounds as 1H-pyrrolo [2,3-b] pyridines [18], isoxazolones [19], ethylated thiazole-triazole acetamides [20], indoles [21] and cinnolines derivatives [22]. Interesting elastase inhibitors include cyclic depsipeptides such as loggerpeptins A-C and molassamide isolated from marine cyanobacteria, which in addition to their high inhibitory activity against neutrophil elastase also inhibited the migration of highly invasive MDA-MB-231 breast cancer cells [23].

An interesting pharmacophore in the search for compounds with different biological activities is phthalimide. The best-known compound containing this moiety is Thalidomide, an agent used in the 1960s as a sedative and antiemetic for pregnant women, withdrawn from the market due to teratogenic effects [24]. However, it is a great example of drug repositioning, as subsequent studies on thalidomide have led to the discovery of its pleiotropic biological activity. Thalidomide has been shown to be an inhibitor of peptidases, glucosidase and COX, it decreases the activity of the important pro-inflammatory cytokine tumor necrosis factor-alpha (TNF-alpha), and also exhibits antiangiogenic and proapoptotic activity. The discovered potent antineoplastic and immunomodulatory properties have led to the application of Thalidomide and its derivatives in the therapy of multiple myeloma, erythema nodosum, as well as in studies on the use of this compound in other types of cancer, including haematological ones [25]. Such broad biological activity of Thalidomide prompts a closer look at its chemical structure. It consists of two rings, glutarimide and phthalimide, the latter of which is an important pharmacophore used in drug design. Due to its hydrophobic nature, it has the ability to cross biological membranes under in vivo conditions. Phthalimide derivatives have been shown to exhibit antibacterial [26], antiparasitic [27], antifungal [28] and analgesic [29] activities. Compounds containing a phthalimide group in the molecule also show potent anticancer activity against various types of tumor cells, i.e., prostate [30,31], breast [31,32] or liver cancer cells [31,33].

Another biologically important pharmacophore is thiazole ring, found in compounds with anticancer [34,35,36], antimicrobial [37], antituberculosis [38], anticonvulsant [39,40], and anti-Toxoplasma gondii activity [41,42]. Thiazole derivatives were presented as inhibitors of important protein kinases such as EGFR, VEGF and PI3Ks [43], and SARS-CoV 3CL protease [44].

Our previous studies showed that dichloro-substituted phthalimido-thiazole derivatives showed high antitumor activity against MV4-11 and A549 with IC_50_ values in the range of 5.56–16.10 µM. They also showed the ability to inhibit porcine pancreatic elastase, with IC_50_ values in the range of 32.10–93.90 µM [45]. 

In this study, we report the synthesis of new phthalimide-thiazole derivatives and investigate them as new human neutrophil elastase (HNE) inhibitors using spectrofluorimetric and computational methods. The main core of the molecule contains various substituents that are intended to modify the electronic properties and geometry of the molecules. The main compounds were tested for their antitumor activity against four human cancer cells lines, namely biphenotypic B myelomonocytic leukemia MV4-11, human lung carcinoma A549, human breast adenocarcinoma MDA-MB-231 and human bladder translational cell carcinoma UMUC-3, and their cytotoxicity was tested using normal mouse fibroblast (BALB/3T3). Finally, chemical stability of the key compound was also investigated in an aqueous phosphate buffer simulating physiological conditions using the spectrophotometric method.

## 2. Results and Discussion

### 2.1. Chemical Synthesis

**Scheme 1:**
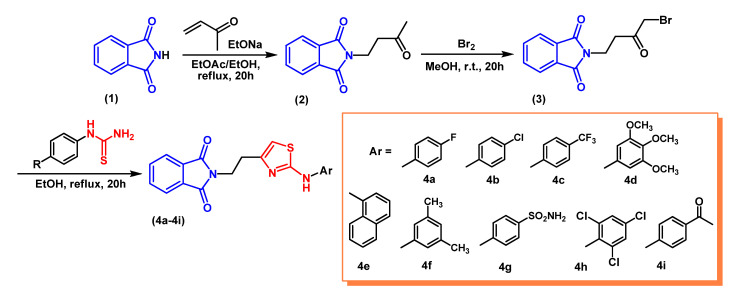
Synthesis of the target phthalimide-thiazoles **4a**–**4i**.

The synthetic pathway leading to compounds **4a**–**4i** is shown in Scheme 1. In the first step, phthalimide **1** was reacted with methyl vinyl ketone in the presence of sodium ethoxide as a catalyst to obtain 2-(3-oxobutyl)phthalimide **2**. In the next step ketone was brominated with bromine in methyl alcohol to give 2-(4-bromo-3-oxobutyl)phthalimide **3** [45,46,47]. In the last step using the Hantzsch reaction of bromoketone **3** with various thioureas, ten final compounds **4a**–**4i** were obtained. The newly synthesized compounds were identified using spectroscopic methods, such as ^1^H NMR (700 MHz), ^13^C NMR (100 MHz) and ESI-HRMS analysis [see Supplementary Materials]. The ^1^H NMR spectra of compounds **4a**–**4i** have four characteristic signals: two triplets derived from methylene groups at about 2.90 ppm and 3.90 ppm, thiazole-5H proton singlet at about 6.50 ppm and singlet of the NH group at 9.45–10.44 ppm. The structure of the newly synthesized compounds was also confirmed using ^13^C NMR spectra. The most characteristic signals were found at about 30 ppm and 37 ppm derived from the two methylene groups, and at about 105 ppm corresponding to the CH of the thiazole ring. The key signals are also those from the two carbonyl groups of the phthalimide ring at about 168 ppm.

### 2.2. Elastase Inhibitory Activity and Kinetic Analysis

The study of human neutrophil elastase inhibition by compounds **4a**–**4i** was carried out using a spectrofluorimetric method. The results of the study are shown in Table 1. Ursolic acid was used as the reference inhibitor. All newly synthesized phthalimide derivatives showed the ability to inhibit neutrophil elastase. The best inhibitory activity was shown by compounds with 4-trifluoromethyl (**4c**), 4-naphthyl (**4e**) and 2,4,6-trichloro (**4h**) substituents in the phenyl ring with IC_50_ values of 12.98 µM, 15.58 µM and 16.62 µM, respectively. These values are only two to three times higher than the IC_50_ value for reference inhibitor—ursolic acid, IC_50_ 5.32 µM. Compounds containing 4-COCH_3_ (**4i**), 3,4,5-triOMe (**4d**) and 4-fluorophenyl (**4a**) substituents also proved to be good elastase inhibitors, with IC_50_ values in the range of 22.92–29.35 µM. The weakest inhibitory activity among the **4a**–**4i** derivatives was shown by compounds **4f** and **4g**, with IC_50_ 49.88 and 64.89 µM, respectively. It can be seen that the HNE inhibitory activity increases in a series of compounds **4a** and **4c** containing 4-fluorophenyl and 4-trifluoromethylphenyl groups, probably due to an increased electron-acceptor or steric effect. Moreover, the same relationship can be seen for derivatives **4b** and **4h** containing chlorophenyl and 2,4,6-trichlorophenyl groups and for derivatives **4b** and **4a** containing chlorophenyl and fluorophenyl groups. 

**Figure 1:**
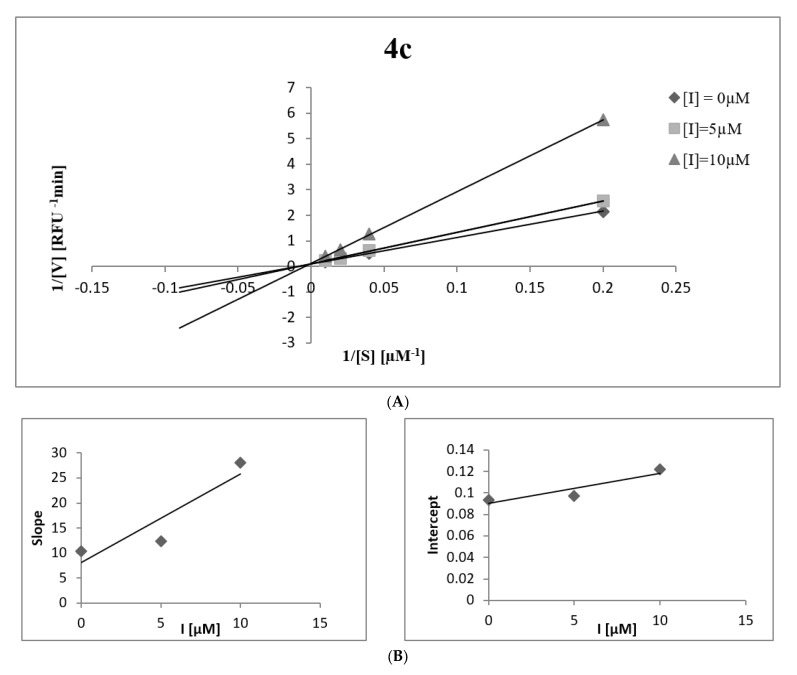
(**A**) Lineweaver–Burk plots for elastase inhibition for compound **4c**. (**B**) The secondary plots of the slope and the intercept of the straight lines versus the concentration of compound **4c**.

To better understand the mechanism of inhibition of human neutrophil elastase by the newly synthesized compounds, kinetic studies using double-reciprocal plots of Lineweaver–Burk and Dixon analysis were performed. The most active compound **4c** was selected for this study. The results are shown in Table 2 and Figure 1. Compound **4c** has been identified as an elastase inhibitor characterized by a mixed type of action. This means that the compound can inhibit the enzyme through two different mechanisms, depending on whether it has a higher affinity for the free enzyme (type I) or the enzyme-substrate complex (type II). To determine which inhibition model characterizes compound **4c**, the appropriate inhibition constants K_i_ and K_is_ were determined using secondary plots of K_m_/V_max_ and 1/V_max_ versus inhibitor concentration. In the case of compound **4c**, the K_i_ constant is smaller than the K_is_ constant, indicating that the compound has a higher affinity for the free enzyme than the enzyme-substrate complex, showing mixed type I inhibition.

**Table 1:** Elastase inhibitory activity of phthalimide-thiazoles **4a**–**4i** compared to standard inhibitor.

Compound	IC_50_ ± SD [µM]
**4a**	29.35 ± 3.13
**4b**	46.51 ± 4.85
**4c**	12.89 ± 0.98
**4d**	22.92 ± 1.59
**4e**	15.58 ± 0.16
**4f**	49.88 ± 0.98
**4g**	64.89 ± 7.75
**4h**	16.62 ± 2.06
**4i**	25.60 ± 1.78
**Ursolic acid**	5.32 ± 0.68

**Table 2:** Kinetic analysis of mechanism of elastase inhibition by compound **4c**.

Compound	Dose [µM]	V_max_	K_m_	Inhibition Type	K_i_ [µM]	K_is_ [µM]
**4c**	5	10.29	127.27	mixed	4.54	32.18
	10	8.22	230.63			

### 2.3. Antiproliferative Activity

The newly synthesized compounds were tested for their antiproliferative activity against four cancer cell lines: human biphenotypic B myelomonocytic leukemia (MV4-11), lung carcinoma (A549), human breast adenocarcinoma (MDA-MB-231), urinary bladder carcinoma (UMUC-3), and normal mouse fibroblast (BALB/3T3) cells. The results are shown in Table 3. It can be seen that compounds **4b**, **4d** and **4f** containing 4-chloro, 3,4,5-trimethoxy and 3,5-dimethyl substituents in the phenyl ring have high antiproliferative activity against human cancer cells lines MV4-11, with IC_50_ in the range of 8.21–13.19 µM. The same series of compounds **4b**, **4d** and **4f** extended by the compound **4c** with trifluoromethyl group have also high antiproliferative activity against A549 cell lines, with IC_50_ in the range of 13.99–25.57 µM. Additionally, compounds **4g** with sulfonamide group showed high activity against MDA-MB-231 and UMUC-3 with IC_50_ values of 9.66 and 19.81µM, respectively. Moreover, compound **4e** containing a naphthyl ring in its structure showed good antiproliferative activity against MV4-11 and A549 cancer cell lines, with IC_50_ of 33.25 and 41.07 µM, respectively. The less potent of the series for this cancer cell line were compounds **4a**, **4h** and **4i**, with IC_50_ values in the range of 44.45–120.05 µM. Next, we decided to calculate the selectivity index (SI) by comparing the cytotoxic activity (IC_50_) of compounds against the normal fibroblasts BALB/3T3 with the cytotoxic activity (IC_50_) of cancer cell lines (Table 4). The selectivity indexes for phthalimide derivatives **4a**–**4i** against MV4-11 and A-549 cancer cell lines were in general much higher than for MDA-MB-231 and UMUC-3 cancer cells. The highest selectivity index for MV4-11 and A549, SI values in the range of 5.42–7.68, was found for the compounds **4b**, **4c**, **4d** and **4f**. Additionally, with the exception of compounds **4a** and **4g**, the SI values are greater than 1, which proves that the obtained phthalimides are more effective against cancer cells than toxic against normal cells.

### 2.4. Compounds Stability

**Figure 2:**
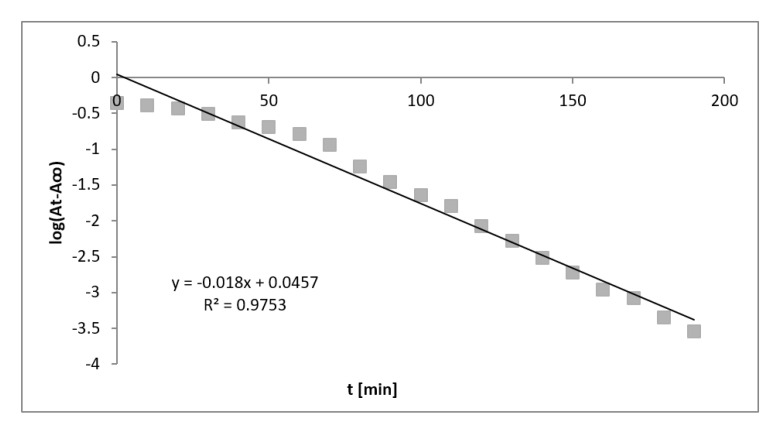
Semilogarithmic plot used to determine rate constants for spontaneous hydrolysis of compound **4c**.

**Figure 3:**
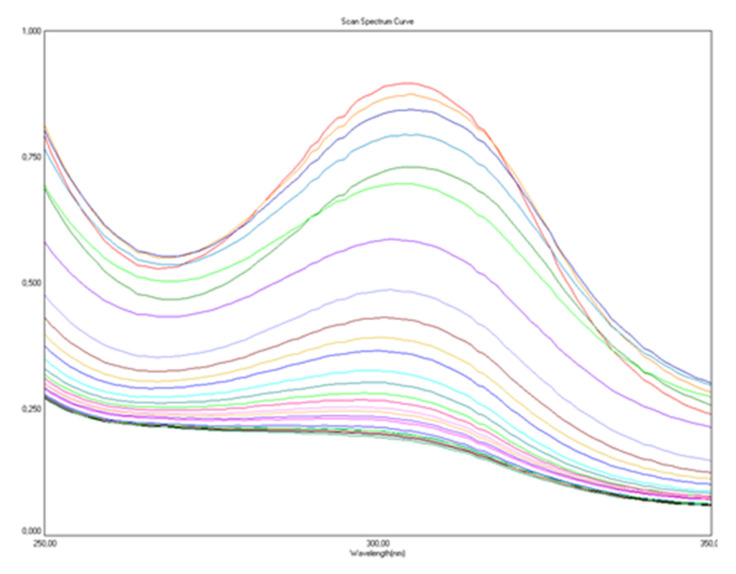
The changes in absorbance spectra of compound 4c during spontaneous hydrolysis. Spectrum was monitored for 250 min with 10 min intervals in 0.05 M phosphate buffer (pH 7.3, 25 °C).

The stability of new substances is a key parameter that determines the effectiveness of a drug as well as its safety. Therefore, the most active phthalimide derivative **4c** was assessed for chemical stability in an aqueous phosphate buffer at pH 7.3 simulating physiological conditions using spectrophotometric method (Table 5). In the tested phosphate buffer, a decrease in the maximum absorbance at 305 nm was observed over time, which indicates a spontaneous hydrolysis of the compound **4c** (Figure 2 and Figure 3). The calculated half-life (t_1/2_) value is 38.5 min, which proves the high stability of the compound under physiological pH conditions.

**Table 5:** Half-life (t1/2) for the spontaneous hydrolysis of selected compound **4c**.

Phthalimide	λ [nm]	k [min^−1^]	t_1/2_ [min]
**4c**	305	0.018	38.5

### 2.5. Molecular Docking Study

The molecular docking study was used to analyses the influence of pharmacophore group on the activity of the studied compounds. According to literature reports [48] the elastase inhibitors can be bound to protein by the formation of covalent bond between Ser195 and the ligand group capable to participate in the condensation reaction. Subsequently, the ligand molecule may undergo a decomposition into two fragments. Paradoxically, event in such case, the covalent docking procedure, involving the whole ligand molecule does not seem suitable for predicting the inhibition properties due to the following facts: (i) the low-energy ligand poses do not necessarily correspond to the conformations that facilitates the prospective reaction with serine, thus, they may be chemically meaningless; (ii) after the ligand decomposition, the covalently bound ligand fragment may be common for all considered compounds, thus, performing covalent docking would not produce any distinguishable results. On the other hand, one of our previous results indicate that standard docking produces the trend in binding energies which is in agreement with the experimental values of IC_50_ [45]. Previously [49], we have applied the standard, non-covalent docking with the so-called ‘logical constraint’, responsible to reflect the spatial requirements necessary to consider the ligand-Ser195 reaction. This resulted in a reasonable agreement of the binding energies with the experimentally-inferred IC_50_ values but also provided a consistent picture of the ligand-receptor pattern of interactions, common for the whole considered group of compounds. In the initial stages of the current study, we planned to apply the ‘logical constraint’-based procedure, analogous to that described in more detail in ref. [49]. In short, such a procedure relies on introducing the cutoff-based criteria and ignoring all the docking results (structures) in which the distance between Ser195 sidechain and the reaction site within the ligand molecule is larger than 0.3 nm. Here, the prospective reaction site was defined as the two oxygen atoms of the isoindoline-1,3-dione moiety of the ligand molecule. However, during analysis of the obtained docking poses, it was concluded that all the low energy structures already fulfill this additional distance-based condition and no poses corresponding to more favorable ligand-protein interactions and some other arrangements in the binding cavity were observed. This indicates that the mechanism of binding is associated either with the formation of a covalent bond involving Ser195 or at least with creating the network of ligand–protein interactions compatible with early stages of such processes.

**Figure 4:**
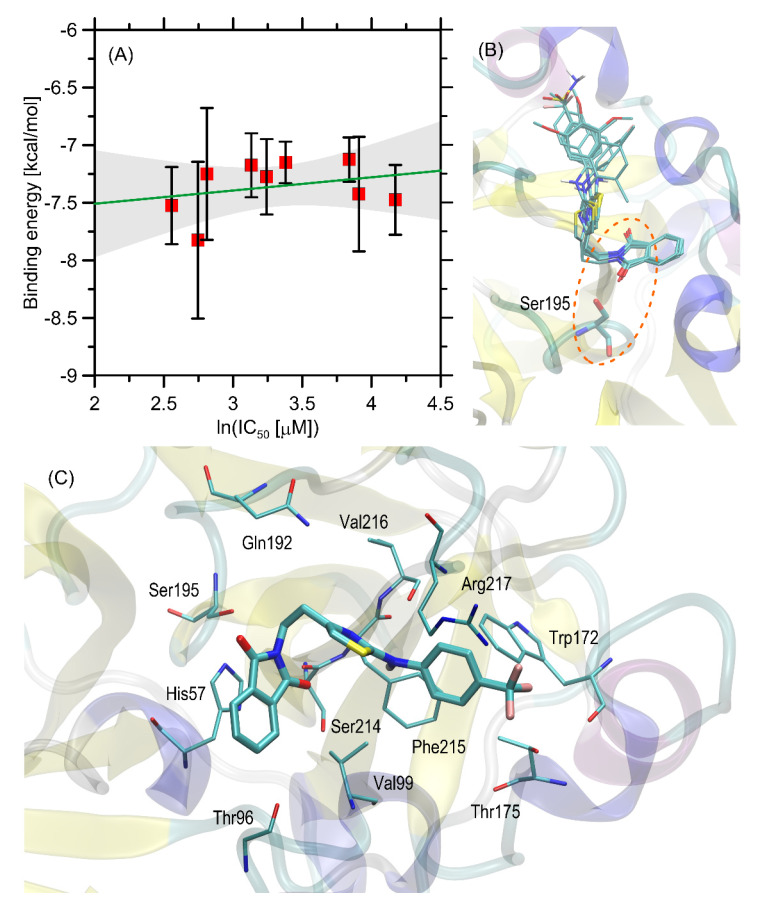
(**A**) The correlation between the calculated binding energies, averaged over the five elastase structures available in the PDB database and the corresponding (experimental) values of IC_50_ (recalculated as log(IC_50_)). The horizontal bars represent the standard deviation values within the set of binding energies. The green solid line represents the linear regression (with 95% confidence intervals filled in grey) (*R* = 0.41; *p* < 0.27). (**B**) The superposed positions of all studied ligands (stick representation, colored by atom type) in the binding cavity of the 1bma structure of elastase. All these positions were identified as the optimal ones during the standard (non-covalent) docking procedure. The maximal distance of oxygen atoms at the isoindoline-1,3-dione group and Ser195 does not exceed 0.3 nm. (**C**) The location of the **4c** ligand molecule bound to the 1bma structure of elastase and found in the docking procedure. The ligand molecule and all the closest amino-acid residues are represented by thick and thin sticks, respectively. The description of the interaction types is given in the text.

The binding energies found during docking simulations are graphically illustrated in Figure 4A. Each of the final energy values was averaged over the set of poses exhibiting the same structural orientation in the binding cavity, as confirmed by separate RMSD calculations and visual inspection (Figure 4B). All the obtained ligand-protein interaction energies display similar magnitude, varying in the range of ~−7.8–−6.8 kcal/mol. Such a small scatter of ligand–protein interaction energies across the studied set of compounds (1.0 kcal/mol) can be explained in terms of their high structural similarity of the studied compounds combined by extremely close locations and orientations exhibited by them when interacting with binding cavity. 

Although the correlation between ln(IC_50_) values and the determined binding energies is not as good (R = 0.41, *p* < 0.27) as those obtained in our previous studies [45,49], some binding trends are reflected correctly. For instance, the two most potent compounds (**4c** and **4e**) display the most and second most favourable binding energies. At the same time one can observe that compounds exhibiting the highest IC_50_ values (**4g** and **4h**) are not correctly identified in the calculations. In fact, ignoring these two data points results in a significant improvement of the statistical parameters that describe the above-mentioned correlation (R = 0.72, *p* < 0.04). There exist several distinct factors that may be responsible for that, for instance: (i) the large scatter of binding energies across the set of considered PDB structures (standard deviation is of magnitude of broadness of the data set values); (ii) the small scatter of both the experimental and theoretical values; (iii) the influence of mechanisms that cannot be reflected by classical force field-based docking, i.e., the processes related to the electronic structure of ligand, essential for hypothetical reaction with Ser195. 

In our previous paper [49], we concluded that a good correlation of the binding energies with the IC_50_ values speaks for dependence of the inhibition properties on the stage of ligand alignment in the binding cavity before the subsequent reaction with Ser195. Here, the analogous correlation is not so obvious. Therefore, it is hard to draw a similar, unmistakable conclusion. Nevertheless, the consistent arrangement of the lowest-energy ligand poses, common for all considered compounds and all protein structures support the hypothesis about the contribution of Ser195 in covalent ligand binding. 

The results of the docking studies were also analysed with respect to the structural/mechanistic protein-ligand interaction pattern that is essential for interpretation of the obtained binding energy values and identifying the physical basis responsible for ligand binding. The summary given below relies on analysing the ligand–protein contacts that take place if the distance between any corresponding atom pair is smaller than the arbitrarily accepted value of 0.4 nm. The provided description can be related to all the studied compounds due to their nearly identical orientations in the binding cavity (Figure 4B). The accuracy limits in this context result mainly from the molecular topology of the ligand, i.e., different chemical character of a single moiety). The graphical illustration of the docking results (on example of 4c) is given in Figure 4C. Note that the analysis below concerns only the non-covalent ligand-protein interactions and does not include the hypothetical, subsequent steps involving covalent bonding with Ser195. The network of interactions responsible for distinguishing between potencies of particular compounds is created by Trp172, Thr175, Phe215 and Arg217. The limiting, aromatic substituents of all compounds always maintain contact with Phe215 and Trp172 via attractive π-π or CH-π interactions. The type of interactions with Thr175 varies on the character of substituent and may concern either polar or non-polar interactions, dependent of the character of moieties located in this region of ligand molecule. Interactions with Arg217 occur with both the thiazole ring and limiting, aromatic moiety (as shown in Figure 4C and according to description below). The Arg217 sidechain is the most flexible among all sidechains considered explicitly during docking and can exhibit various, conformationally-diverse contacts with those two regions of the ligand molecule. Nearly the same network of contacts, also distinguishing between inhibition properties, has been identified for other group of elastase inhibitors in our previous study [49]. The physical-chemical character of interactions formed by this crucial substituent and neighboring residues is not uniform and involves e.g., hydrogen bonding donation and acceptance, CH-π and π-π stacking, therefore, the detailed interpretation of the dependence of the moiety type on the compound potency is not straightforward. Basing on the case of the two most potent compounds (for which the prediction of docking results agrees with the experimental results) it can be concluded that the increased contribution of the CH-π and π-π stacking (compound **4e**) has a positive effect on the favorability of the ligand-protein interactions. The same can be said about introduction of fluorine atoms (compound **4c**), which, in theory, allows for creation of halogen bonds with Trp172 and Thr175. However, larger number of fluorine atoms at the aromatic substituent (compound **4e**) apparently results in unfavorable steric restrictions and increase of the interaction energy. The remaining ligand-protein interactions concern the polar interactions of Arg217 with thiazole ring (hydrogen bonding) and CH-π stacking involving also the thiazole ring and Val99 sidechain. The thiazole ring also maintains contact with backbone fragment of Val216, but it seems to be only an opportunistic consequence of other, stronger interactions, imposing the corresponding conformational arrangement. The aliphatic linker connecting thiazole ring with the isoindoline-1,3-dione group maintains contact with Gln192. Again, this interaction does not seem to be crucial in the context of ligand-protein interactions strength. The part of the ligand molecule that contains the isoindoline-1,3-dione moiety is especially crucial for occurrence of the possible reaction with Ser195. The attractive, hydrogen bonding-mediated interactions are always observed in the case of one of the oxygen atoms in the isoindoline-1,3-dione group and neighboring Ser195 sidechain. However, the associated O-O distance is slightly larger (0.27) than it was observed in the case of our previous study and analogous contact [49]; this is the consequence of increase dimension of the group interacting with Ser195. This contact and arrangement of the isoindoline-1,3-dione group is stabilized by the CH-π and π-π stacking with neighboring His57 sidechain. The vicinity of the two other residues (Thr96 and Ser214) seems to be only an opportunistic consequence of other protein-ligand interactions discussed above.

## 3. Materials and Methods

### 3.1. Chemistry

#### 3.1.1. 2-(3-Oxobutyl)phthalimide (**2**)

3-Buten-2-one (7.0 g, 100.0 mmol) was added to a stirred solution of phthalimide (**1**) (14.70 g, 100.0 mmol) in ethyl acetate (100 mL) and then sodium ethoxide (0.27 g) in absolute ethyl alcohol (25 mL) was added. The reaction mixture was stirred at room temperature for 2 h, and next under reflux until a clear solution was obtained. Heating was continued for additional 2 h, next solvent was evaporated, and the crude product was crystallized from ethyl alcohol to yield 18.0 g (83%); mp 108–110 °C [45,46,47]; eluent: dichloromethane/methanol (95:5); R_f_ = 0.55. ^1^H NMR (400 MHz, DMSO-d_6_), δ(ppm): 2.12 (s, 3H, CH_3_); 2.84 (t, 2H, CH_2_, J = 7.2 Hz); 3.76 (t, 2H, CH_2_, J = 7.4 Hz); 7.81–7.87 (m, 4H, 4CH_Ar_). ^13^C NMR (100 MHz, DMSO-d_6_), δ(ppm): 30.11 (CH_3_); 33.04 (CH_2_); 41.29 (CH_2_); 123.35 (2C); 132.02 (2C); 134.74 (2C); 168.05 (2C); 206.77 (C).

#### 3.1.2. 2-(4-Bromo-3-oxobutyl)phthalimide (**3**)

4-*N*-Phtalimido-2-butanone (**2**) (7.0 g, 32.23 mmol) was dissolved in methanol (100 mL). A solution of bromine (1.65 mL, 32.23 mmol) was added dropwise over 30 min at 0 °C. The reaction mixture was stirred at room temperature for 15 h. The separate precipitate was collected by filtration and dried under vacuum to yield 8.33 g (87%); mp 115–116 °C [45,46,47]; eluent: dichloromethane/methanol (95:5); R_f_ = 0.84. ^1^H NMR (400 MHz, DMSO-d_6_), δ(ppm): 3.00 (t, 2H, CH_2_, J = 7.1 Hz); 3.82 (t, 2H, CH_2_, J = 7.1 Hz); 4.37 (s, 2H, CH_2_); 7.81–7.89 (m, 4H, 4CH_Ar_). ^13^C NMR (100 MHz, DMSO-d_6_), δ(ppm): 33.06 (CH_2_); 37.09 (CH_2_); 37.88 (CH_2_); 123.38 (2C); 132.04 (2C); 134.75 (2C); 168.04 (2C); 200.09 (C).

#### 3.1.3. 2-(2-(2-(4-Fluorophenylamino)thiazol-4-yl)ethyl)isoindoline-1,3-dione (**4a**) Typical Procedure

2-(4-Bromo-3-oxobutyl)phthalimide (**3**) (0.296 g, 1.0 mmol) was added to a stirred solution of 4-fluorophenylthiourea (0.17 g, 1 mmol) in absolute ethyl alcohol (20 mL). The reaction mixture was stirred under reflux for 20 h. Next, the reaction mixture was added to water (50 mL) and neutralized with NaHCO_3_ solution. The separate precipitate was collected by filtration and dried under vacuum over P_2_O_5_ to afford the desired product: 0.24 g (66%); mp 153–154 °C; eluent: dichloromethane/methanol (95:5); R_f_ = 0.63. ^1^H NMR (400 MHz, DMSO-d_6_), δ(ppm): 2.86 (t, 2H, CH_2_, J = 6.3 Hz); 3.85 (t, 2H, CH_2_, J = 6.3 Hz); 6.51 (s, 1H, CH_thiazole_); 6.94 (t, 2H, 2CH_Ar_, J = 8.4 Hz); 7.46 (q, 2H, 2CH_Ar_, J = 9.1 Hz); 7.77–7.79 (m, 2H, 2CH_Ar_); 7.80–7.83 (m, 2H, 2CH_Ar_); 10.03 (s, 1H, NH). ^13^C NMR (100 MHz, DMSO-d_6_), δ(ppm): 30.23 (CH_2_); 37.82 (CH_2_); 103.83 (C_thiazole_); 115.61 (d, 2C, J = 22.5 Hz); 118.51 (d, 2C, J = 7.6 Hz); 123.45 (2C); 132.12 (2C); 134.76 (2C); 138.09 (C); 149.51 (C); 157.03 (d, C, J = 235.9 Hz); 163.71 (C); 168.18 (2C=O). ESI-HRMS (*m*/*z*) calculated for C_19_H_15_FN_3_O_2_S: 368.0869 [M + H]^+^. Found 368.0862.

#### 3.1.4. 2-(2-(2-(4-Chlorophenylamino)thiazol-4-yl)ethyl)isoindoline-1,3-dione (**4b**)

Yield: 0.29 g, 76%, (dichloromethane/methanol (95:5); R_f_ = 0.52); mp 165–167 °C; ^1^H NMR (400 MHz, DMSO-d_6_), δ(ppm): 2.88 (t, 2H, CH_2_, J = 7.0 Hz); 3.85 (t, 2H, CH_2_, J = 6.3 Hz); 6.56 (s, 1H, CH_thiazole_); 7.12 (d, 2H, 2CH_Ar_, J = 9.8 Hz); 7.47 (d, 2H, 2CH_Ar_, J = 8.4 Hz); 7.76–7.79 (m, 2H, 2CH_Ar_); 7.79–7.82 (m, 2H, 2CH_Ar_); 10.16 (s, 1H, NH). ^13^C NMR (100 MHz, DMSO-d_6_), δ(ppm): 30.20 (CH_2_); 37.82 (CH_2_); 104.33 (C_thiazole_); 118.45 (2C); 123.44 (2C); 124.54 (C); 128.85 (2C); 132.11 (2C); 134.71 (2C); 140.44 (C); 149.60 (C); 163.27 (C); 168.17 (2C=O). ESI-HRMS (*m*/*z*) calculated for C_19_H_15_ClN_3_O_2_S: 384.0573 [M + H]^+^. Found 384.0569.

#### 3.1.5. 2-(2-(2-(4-(Trifluoromethyl)phenylamino)thiazol-4-yl)ethyl)isoindoline-1,3-dione (**4c**)

Yield: 0.31 g, 73%, (dichloromethane/methanol (95:5); R_f_ = 0.62); mp 170–172 °C. ^1^H NMR (400 MHz, DMSO-d_6_), δ(ppm): 2.91 (t, 2H, CH_2_, J = 7.0 Hz); 3.86 (t, 2H, CH_2_, J = 7.0 Hz); 6.65 (s, 1H, CH_thiazole_); 7.38 (d, 2H, 2CH_Ar_, J = 8.4 Hz); 7.60 (d, 2H, 2CH_Ar_, J = 8.4 Hz); 7.74–7.76 (m, 2H, 2CH_Ar_); 7.79–7.80 (m, 2H, 2CH_Ar_); 10.44 (s, 1H, NH). ^13^C NMR (100 MHz, DMSO-d_6_), δ(ppm): 30.06 (CH_2_); 37.86 (CH_2_); 105.29 (C_thiazole_); 116.56 (2C); 123.44 (2C); 126.34 (q, C, J = 7.65 Hz); 126.38 (C); 126.42 (C); 132.16 (2C); 134.72 (2C); 144.71 (C); 149.77 (2C); 162.78 (C); 168.21 (2C=O). ESI-HRMS (*m*/*z*) calculated for C_20_H_15_F_3_N_3_O_2_S: 418.0837 [M + H]^+^. Found 418.0831.

#### 3.1.6. 2-(2-(2-(3,4,5-Trimethoxyphenylamino)thiazol-4-yl)ethyl)isoindoline-1,3-dione (**4d**)

Yield: 0.26 g, 58%, (dichloromethane/methanol (95:5); R_f_ = 0.63); mp 80–82 °C. ^1^H NMR (400 MHz, DMSO-d_6_), δ(ppm): 2.85 (t, 2H, CH_2_, J = 7.7 Hz); 3.56 (s, 3H, CH_3_); 3.75 (s, 6H, 2CH_3_); 3.89 (t, 2H, CH_2_, J = 7.7 Hz); 6.52 (s, 1H, CH_thiazole_); 6.98 (s, 2H, 2CH_Ar_); 7.76–7.78 (m, 2H, 2CH_Ar_); 7.79–7.83 (m, 2H, 2CH_Ar_); 9.99 (s, 1H, NH). ^13^C NMR (100 MHz, DMSO-d_6_), δ(ppm): 30.57 (CH_2_); 37.56 (CH_2_); 56.09 (2CH_3_); 60.57 (CH_3_); 95.20 (2C); 103.76 (C_thiazole_); 123.44 (2C); 132.09 (2C); 132.28 (C); 134.74 (2C); 137.88 (C); 149.10 (C); 153.37 (2C); 163.73 (C); 168.21 (2C=O). ESI-HRMS (*m*/*z*) calculated for C_22_H_22_N_3_O_5_S: 440.1280 [M + H]^+^. Found 440.1274.

#### 3.1.7. 2-(2-(2-(Naphthalen-1-ylamino)thiazol-4-yl)ethyl)isoindoline-1,3-dione (**4e**)

Yield: 0.27 g, 67%, (dichloromethane/methanol (95:5); R_f_ = 0.48); mp 161–162 °C. ^1^H NMR (400 MHz, DMSO-d_6_), δ(ppm): 2.87 (t, 2H, CH_2_, J = 7.7 Hz); 3.87 (t, 2H, CH_2_, J = 7.0 Hz); 6.53 (s, 1H, CH_thiazole_); 7.28 (t, 1H, CH_Ar_, J = 7.7 Hz); 7.49–7.51 (m, 2H, 2CH_Ar_); 7.55 (d, 1H, CH_Ar_, J = 8.4 Hz); 7.77–7.79 (m, 2H, 2CH_Ar_); 7.82–7.84(m, 2H, 2CH_Ar_); 7.87–7.89 (m, 1H, CH_Ar_); 8.02 (d, 1H, CH_Ar_, J = 7.0 Hz); 8.22 (t, 1H, CH_Ar_, J = 4.2 Hz); 9.92 (s, 1H, NH). ^13^C NMR (100 MHz, DMSO-d_6_), δ(ppm): 30.39 (CH_2_); 37.80 (CH_2_); 104.36 (C_thiazole_); 116.36 (C); 122.61 (C); 123.21 (C); 123.45 (2C); 125.94 (C); 126.17 (C); 126.33 (C); 126.47 (C); 128.66 (C); 132.12 (2C); 134.39 (C); 134.78 (2C); 137.16 (C); 149.33 (C); 165.75 (C); 168.20 (2C=O). ESI-HRMS (*m*/*z*) calculated for C_23_H_18_N_3_O_2_S: 400.1120 [M + H]^+^. Found 400.1114.

#### 3.1.8. 2-(2-(2-(3,5-Dimethylphenylamino)thiazol-4-yl)ethyl)isoindoline-1,3-dione (**4f**)

Yield: 0.22 g, 58%, (dichloromethane/methanol (95:5); R_f_ = 0.55); mp 60–61 °C. ^1^H NMR (400 MHz, DMSO-d_6_), δ(ppm): 2.18 (s, 6H, 2CH_3_); 2.87 (t, 2H, CH_2_, J = 7.2 Hz); 3.88 (t, 2H, CH_2_, J = 7.6 Hz); 6.49 (s, 1H, CH_Ar_); 6.53 (s, 1H, CH_thiazole_); 7.14 (s, 2H, 2CH_Ar_); 7.77–7.83 (m, 4H, 4CH_Ar_); 9.89 (s, 1H, NH). ^13^C NMR (100 MHz, DMSO-d_6_), δ(ppm): 21.69 (2CH_3_); 30.51 (CH_2_); 37.64 (CH_2_); 103.52 (C_thiazole_); 115.05 (2C); 123.17 (C); 123.41 (2C); 132.09 (2C); 134.76 (2C); 138.23 (2C); 141.57 (C); 149.38 (C); 163.77 (C); 168.18 (2C=O). ESI-HRMS (*m*/*z*) calculated for C_21_H_20_N_3_O_2_S: 378.1276 [M + H]^+^. Found 378.1269.

#### 3.1.9. 4-(4-(2-(1,3-Dioxoisoindolin-2-yl)ethyl)thiazol-2-ylamino)benzenesulfonamide (**4g**)

Yield: 0.23 g, 54%, (dichloromethane/methanol (95:5); R_f_ = 0.23); mp > 260 °C. ^1^H NMR (400 MHz, DMSO-d_6_), δ(ppm): 2.92 (t, 2H, CH_2_, J = 6.8 Hz); 3.89 (t, 2H, CH_2_, J = 6.8 Hz); 6.65 (s, 1H, CH_thiazole_); 7.13 (s, 2H, NH_2_); 7.55–7.58 (m, 4H, 4CH_Ar_), 7.75–7.78 (m, 2H, 2CH_Ar_); 7.78–7.82 (m, 2H, 2CH_Ar_); 10.42 (s, 1H, NH). ^13^C NMR (100 MHz, DMSO-d_6_), δ(ppm): 30.16 (CH_2_); 37.77 (CH_2_); 105.27 (C_thiazole_); 116.22 (2C); 123.42 (2C); 127.27 (2C); 132.07 (2C); 134.83 (2C); 136.04 (C); 144.19 (C); 149.69 (C); 162.81 (C); 168.22 (2C=O). ESI-HRMS (*m*/*z*) calculated for C_19_H_17_N_4_O_4_S_2_: 429.0691 [M + H]^+^. Found 429.0685.

#### 3.1.10. 2-(2-(2-(2,4,6-Trichlorophenylamino)thiazol-4-yl)ethyl)isoindoline-1,3-dione (**4h**)

Yield: 0.37 g, 81%, (dichloromethane/methanol (95:5); R_f_ = 0.39); mp 168–169 °C. ^1^H NMR (400 MHz, DMSO-d_6_), δ(ppm): 2.75 (t, 2H, CH_2_, J = 7.0 Hz); 3.74 (t, 2H, CH_2_, J = 7.0 Hz); 6.48 (s, 1H, CH_thiazole_); 7.63 (s, 2H, 2CH_Ar_); 7.80 (bs, 4H, 4CH_Ar_); 9.54 (s, 1H, NH). ^13^C NMR (100 MHz, DMSO-d_6_), δ(ppm): 29.70 (CH_2_); 37.38 (CH_2_); 123.38 (2C); 129.04 (4C); 132.08 (2C); 134.74 (2C); 165.60 (C); 168.11 (2C=O). ESI-HRMS (*m*/*z*) calculated for C_19_H_13_Cl_3_N_3_O_2_S: 451.9794 [M + H]^+^. Found 451.9791.

#### 3.1.11. 2-(2-(2-(4-Acetylphenylamino)thiazol-4-yl)ethyl)isoindoline-1,3-dione (**4i**)

Yield: 0.24 g, 62%, (dichloromethane/methanol (95:5); R_f_ = 0.38); mp 174–175 °C. ^1^H NMR (400 MHz, DMSO-d_6_), δ(ppm): 2.46 (s, 3H, CH_3_); 2.93 (t, 2H, CH_2_, J = 6.8 Hz); 3.88 (t, 2H, CH_2_, J = 6.8 Hz); 6.67 (s, 1H, CH_thiazole_); 7.53 (d, 2H, 2CH_Ar_, J = 8.8 Hz); 7.69 (d, 2H, 2CH_Ar_, J = 8.8 Hz); 7.75–7.78 (m, 2H, 2CH_Ar_); 7.8–7.83 (m, 2H, 2CH_Ar_); 10.48 (s, 1H, NH). ^13^C NMR (100 MHz, DMSO-d_6_), δ(ppm): 26.72 (CH_3_); 30.13 (CH_2_); 37.88 (CH_2_); 105.48 (C_thiazole_); 115.97 (2C); 123.50 (2C); 129.81 (C); 130.05 (2C); 132.18 (2C); 134.78 (2C); 145.51 (C); 149.76 (C); 162.82 (C); 168.13 (2C=O); 196.14 (C=O). ESI-HRMS (*m*/*z*) calculated for C_21_H_18_N_3_O_3_S: 392.1069 [M + H]^+^. Found 392.1061.

### 3.2. Inhibitory Activity 

#### 3.2.1. Elastase Inhibition Assay

Fluorometric assay for human neutrophil elastase (HNE) inhibition was performed according to the previously presented method with some modifications [50], using N-methoxysuccinyl-Ala-Ala-Pro-Val-7-amido-4-methyl-coumarin (MOSuc-AAPV-AMC, Calbiochem) as substrate. Stock solutions of the tested compounds were prepared in DMSO, the final content of which in the tested sample did not exceed 1%. The reaction was carried out in assay buffer containing 0.1 M HEPES (pH 7.5). Briefly, different concentration of tested compounds was incubated with 20 mU/mL of HNE (Bradford) in 25 °C. The reaction was started by addition of 25 µM elastase substrate to final reaction volume 100 µL and monitored for 30 min at 25 °C on a microplate reader (FP-8500 Spectrofluorometer, JASCO, USA) with excitation and emission wavelengths of 380 nm and 440 nm, respectively. By computing the log of inhibitors concentrations versus the percentage of activity, the IC_50_ values were determined by non-linear regression analysis. Assays were performed in triplicate and data presented as the mean and the standard deviation. Ursolic acid was used as the reference compound.

#### 3.2.2. Kinetic Analysis of the Inhibition of Elastase 

Kinetic studies were conducted in the presence of different concentrations of substrate (5, 25, 50 and 100 µM) and test compound (in the range of 0–10 µM). The reaction conditions were the same as in the HNE inhibition assay and the reaction was monitored for 10 min at 25 °C with the same E_x_ an E_m_ wavelengths. By Lineweaver–Burk plots, kinetic values such as Michaelis–Menten constants and maximum velocity were determined. Next, two inhibition constants for inhibitor binding with free enzyme or enzyme–substrate complex (K_i_ and K_is_) were obtained by plotting the secondary plot of the slopes of the determined straight lines or vertical intercept versus inhibitor concentration [51].

### 3.3. Antiproliferative Activity

Cell preparation, in vitro antiproliferative assay, MTT and SRB cytotoxic tests were performed according to literature [45,52,53,54,55].

### 3.4. Analysis of Compounds Stability

Spontaneous compound hydrolysis studies were performed following the literature procedure [56]. The kinetics hydrolysis of the compounds was evaluated at 25 °C in 0.05 M phosphate buffer, pH 7.3, by measuring changes in absorbance spectra during incubation using a T60U spectrophotometer (PG Instruments). The absorbance (A_t_) at characteristic absorption maxima for each compound was measured at 10 min intervals until there was no further decrease in absorbance (A_ꝏ_). Using these measurements, semilogarithmic plots of the dependence of log(A_t_–A_ꝏ_) on time were prepared, and k’ values were determined from the slope of these plots according to the first order reaction kinetics. Half-conversion times were calculated using equation: t_1/2_ = 0.693/k’.

### 3.5. Molecular Docking Study

The ligand molecules were drawn manually by using Avogadro 1.1.1 [57] and optimized within the UFF force field [58] (5000 steps, conjugate gradient algorithm). The flexible, optimized ligands were docked into the binding pockets of the high-resolution elastase structures found in the following five PDB (rcsb.org) entries: 1bma (X-ray resolution: 0.192 nm), 1hv7 (X-ray resolution: 0.17 nm), 1qnj (X-ray resolution: 0.11 nm), 2de9 (X-ray resolution: 0.13 nm) and 2h1u (X-ray resolution: 0.16 nm). The AutoDock Vina software [59] was applied in all docking simulations. The procedure of docking was carried out within the cuboid region (of dimensions: 20 × 20 × 20 Å^3^) which covered the whole co-crystallized ligand present in the PDB:2h1u structure as well as the closest amino-acid residues that exhibit contact with that ligand. In order to provide sufficient numbers of conformationally-diverse ligand arrangements, the number of possible poses generated during single run was increased to 20, whereas the energy threshold between highest- and lowest-ranked poses was increased to 4 kcal/mol. Apart from that, all the default procedures and algorithms implemented in AutoDock Vina were kept. The predicted binding energies were averaged over all protein structures applied for docking. The more favorable binding mode is associated with the lower binding energy value; only the lowest energy values corresponding to the given ligand were accounted for during subsequent analysis. The visual inspection of the location and orientation of the docked ligands, in order to control the uniformity of the binding pattern was performed. The docking methodology was initially validated by docking simulation of the non-covalently-bound ligand molecule originally included in one of the studied protein structures (i.e., 2h1u). Details are given in our previous work [45].

## 4. Conclusions

The phthalimide-thiazole derivatives with various substituents in the phenyl ring have been synthesized to explore their role in human neutrophil elastase inhibition and antiproliferative activity using spectrofluorimetric, spectrophotometric and computational approaches. The most active compounds containing 4-trifluoromethyl, 4-naphthyl and 2,4,6-trichloro substituents exhibit high HNE inhibitory activity with IC_50_ values of 12.98–16.62 µM. Additionally, compound with 4-trifluoromethyl group showed mixed mechanism of action. Computational investigation resulted in a consistent picture of the ligand-receptor pattern of interactions, common for the whole considered group of compounds in spite of a series of discrepancies observed between the calculated binding energies and the experimentally-inferred IC_50_ values. Moreover, some compounds showed high antiproliferative activity against leukemia, lung, breast, and urinary bladder human cancer cells lines with IC50 values of 8.21 to 25.57 µM. Additionally, spectrophotometric analysis showed that the most active compounds demonstrated high stability under physiological conditions. We believe that analogues containing 4-trifluoromethyl, 4-naphthyl and 2,4,6-trichloro substituents may serve as lead structures to design highly potent HNE inhibitors with good antiproliferative profile.

## Figures and Tables

**Table 3:** Antyproliferative activity of compounds **4a**–**4i** against human biphenotypic B myelomonocytic leukemia (MV4-11), lung carcinoma (A549), human breast adenocarcinoma (MDA-MB-231), human bladder transitional cell carcinoma (UMUC-3) and normal mouse fibroblast BALB/3T3 cells.

Phthalimide Derivatives	IC_50_ ± SD [µM]				
	MV4-11	A549	MDA-MB-231	UMUC-3	BALB/3T3
**4a**	59.55 ± 4.63	50.71 ± 3.32	109.83 ± 24.50	82.31 ± 2.18	74.17 ± 12.25
**4b**	12.22 ± 4.56	18.11 ± 0.94	86.47 ± 10.68	61.74 ± 1.56	66.25 ± 4.17
**4c**	46.57 ± 25.39	13.99 ± 1.56	77.48 ± 13.66	75.20 ± 6.71	84.90 ± 18.21
**4d**	8.21 ± 2.37	23.73 ± 5.23	48.87 ± 13.20	46.08 ± 7.28	46.80 ± 2.05
**4e**	33.25 ± 11.99	41.07 ± 3.13	76.44 ± 11.14	80.01 ± 16.19	102.74 ± 30.70
**4f**	13.19 ± 4.77	25.57 ± 3.63	86.39 ± 12.19	66.47 ± 9.80	101.31 ± 12.19
**4g**	83.32 ± 26.61	NA	9.66 ± 1.40	19.81 ± 1.40	14.35 ± 2.10
**4h**	48.48 ± 21.43	57.05 ± 19.35	NA	120.05 ± 36.00	NA
**4i**	44.45 ± 20.18	62.87 ± 5.75	87.73 ± 21.97	74.06 ± 12.01	98.97 ± 21.20
**cisplatin**	1.43 ± 0.40	5.41 ± 0.60	15.61 ± 2.00	1.73 ± 0.17	8.47 ± 1.96

IC_50_–compound concentration leading to 50% inhibition of cell proliferation was calculated for each experiment separately (experiments were repeated 3–5 times) and data are presented as mean values ± SD, calculated using Prolab-3 system based on Cheburator 0.4 software. NA-not active. cisplatin was used as a positive control [45].

**Table 4:** The calculated selectivity index (SI) of tested compounds. The SI index = IC_50_ of compound in a normal cell line/IC_50_ of the compound in cancer cell line.

Phthalimide Derivatives	SI			
	BALB/3T3/ MV4-11	BALB/3T3/ A549	BALB/3T3/ MDA-MB-231	BALB/3T3/ UMUC-3
**4a**	1.25	1.46	0.68	0.90
**4b**	5.42	3.66	0.77	1.07
**4c**	1.82	6.07	1.10	1.13
**4d**	5.70	1.97	0.96	1.02
**4e**	3.09	2.50	1.34	1.28
**4f**	7.68	3.96	1.17	1.52
**4g**	0.17	-	1.49	0.72
**4h**	-	-	-	-
**4i**	2.23	1.57	1.13	1.33

A beneficial SI > 1.0 indicates a drug with efficacy against cancer cells greater than toxicity against normal cells.

## Data Availability

Not applicable.

## Supplementary Materials

The following supporting information can be downloaded at: https://www.mdpi.com/article/10.3390/ijms24010110/s1.
