# Supplementary material for: Spectrofluorimetric and Computational Investigation of New Phthalimide Derivatives towards Human Neutrophil Elastase Inhibition and Antiproliferative Activity
Source: Int J Mol Sci. 2022 Dec 21;24(1):110. doi: 10.3390/ijms24010110 (PMC9820738; doi:10.3390/ijms24010110)
Supplement: Supplementary file 1 [file ijms-24-00110-s001.zip › ijms-2030564-supplementary.pdf]

## Supporting Information

# Spectrofluorimetric and computational investigation of new phthalimide derivatives towards human neutrophil elastase inhibition and antiproliferative activity

**Beata Donarska[1], Marta Świtalska[2], Joanna Wietrzyk[2], Wojciech Płaziński[3,4], Krzysztof Z. Łączkowski[1]***

*[1]Department of Chemical Technology and Pharmaceuticals, Faculty of Pharmacy, Collegium Medicum, Nicolaus Copernicus University, Jurasza 2, 85-089 Bydgoszcz, Poland,*
*[2]Hirszfeld Institute of Immunology and Experimental Therapy, Polish Academy of Sciences, Rudolfa Weigla 12, 53-114 Wrocław, Poland,*
*[3]Jerzy Haber Institute of Catalysis and Surface Chemistry, Polish Academy of Sciences, Niezapominajek 8, 30-239, Cracow, Poland*
*[4]Department of Biopharmacy, Medical University of Lublin, Chodzki 4a, 20-093 Lublin, Poland*

*Corresponding author: krzysztof.laczkowski@cm.umk.pl

Product **4a**

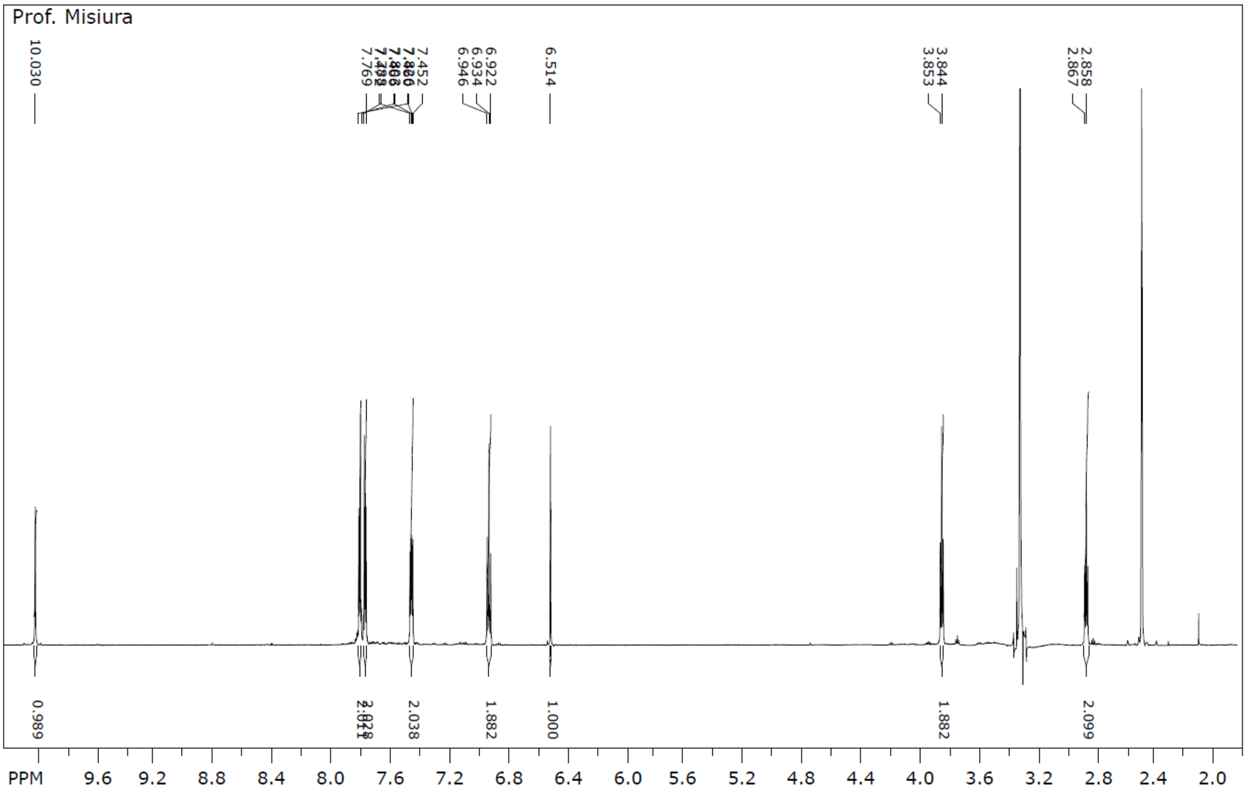

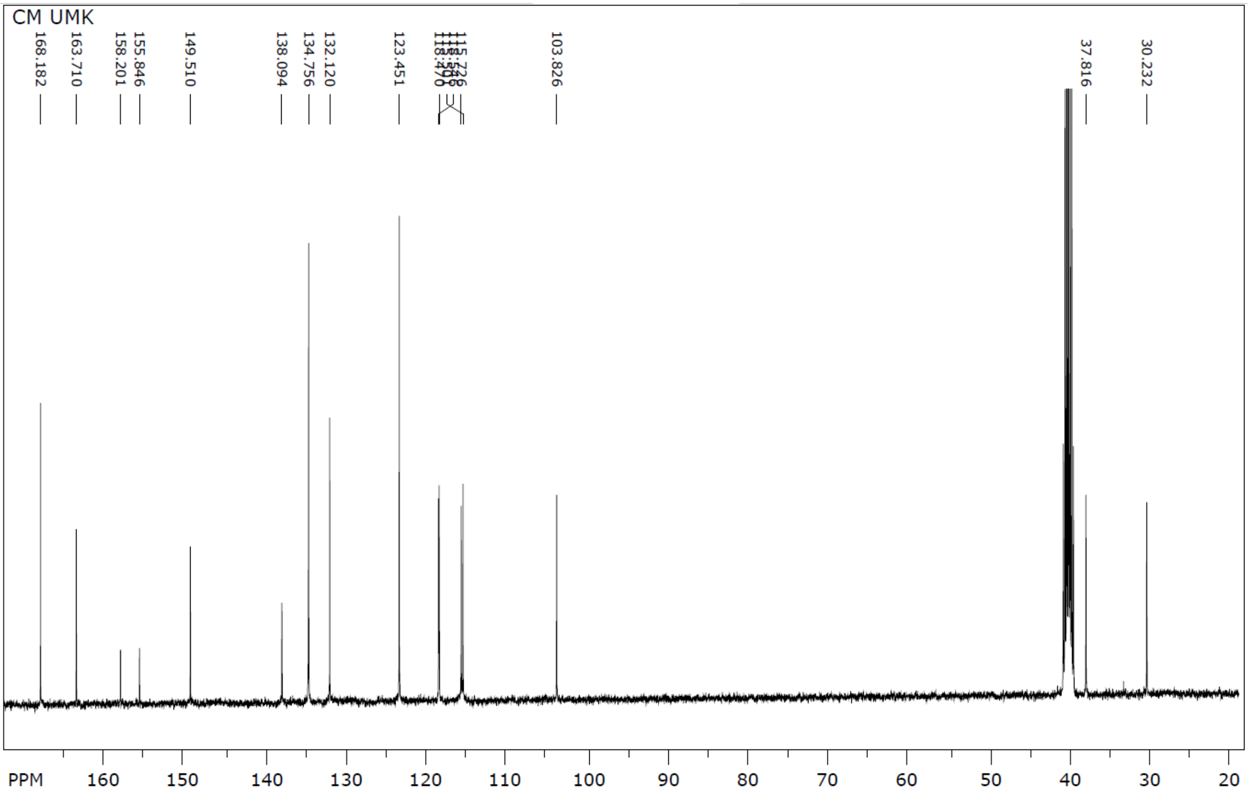
CM UMK
168.182
163.710
158.201
155.846
149.510
138.094
134.756
132.120
123.451
103.826
37.816
30.232
PPM
160
150
140
130
120
110
100
90
80
70
60
50
40
30
20

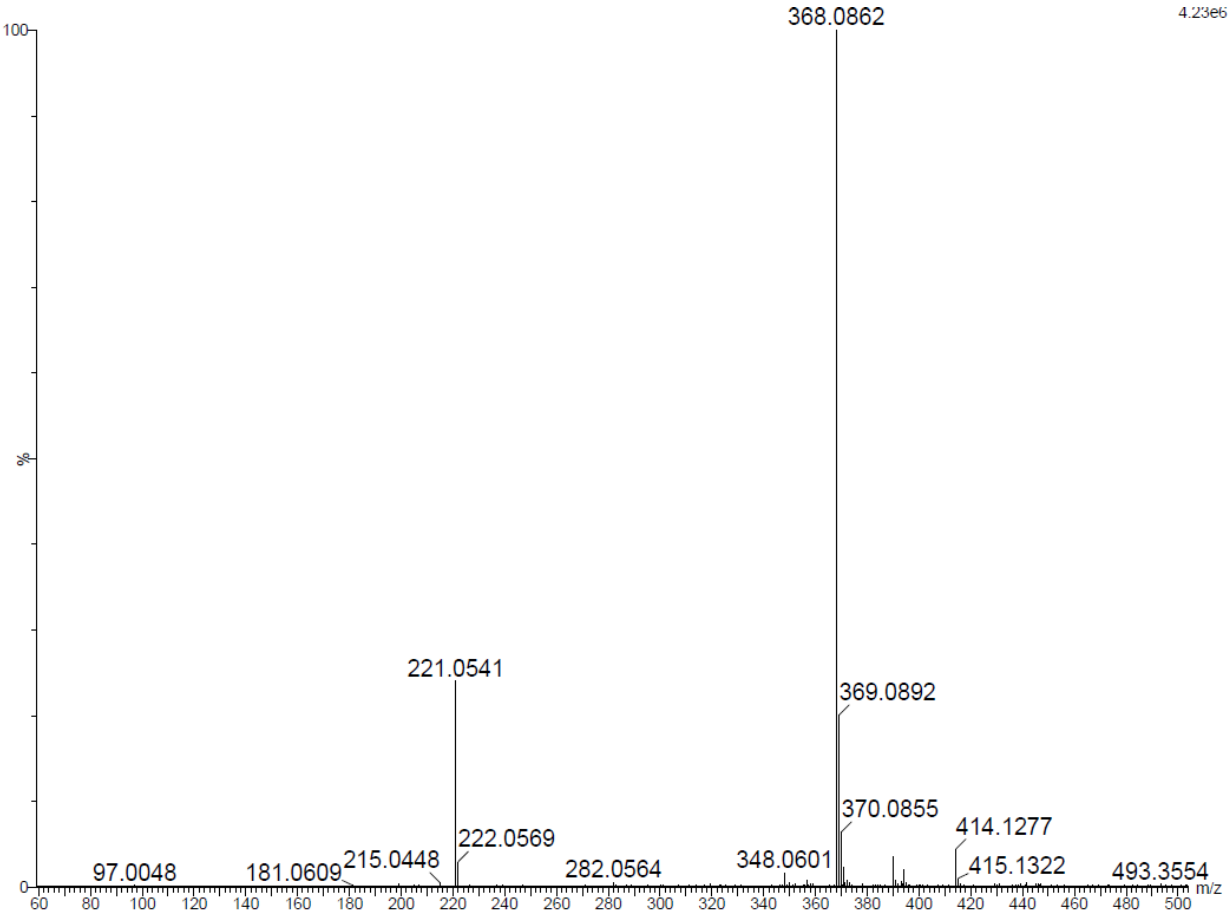
368.0862
4.23e6
100
%
0
221.0541
369.0892
370.0855
414.1277
222.0569
415.1322
97.0048
181.0609
215.0448
282.0564
348.0601
493.3554
m/z
60
80
100
120
140
160
180
200
220
240
260
280
300
320
340
360
380
400
420
440
460
480
500

Product **4b**

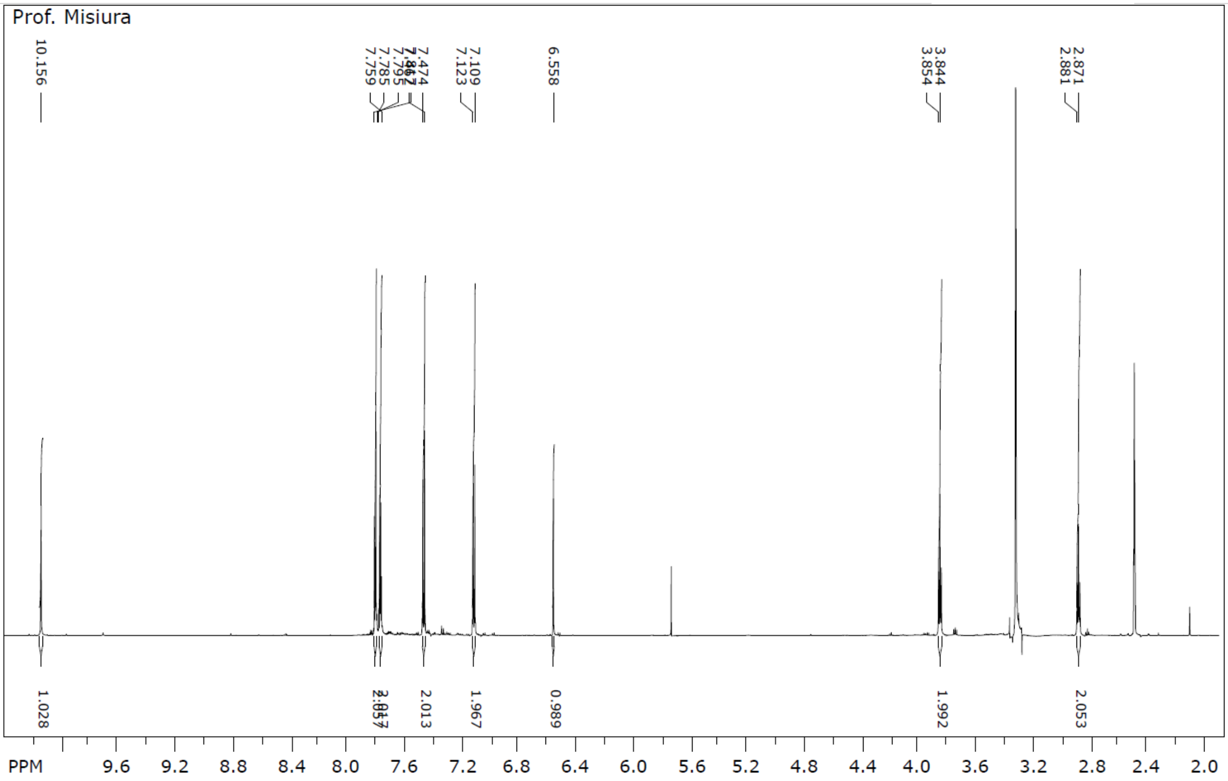

Prof. Misiura
10.156
7.759
7.785
7.474
7.109
7.123
6.558
3.844
3.854
2.871
2.881
1.028
2.013
1.967
0.989
1.992
2.053
PPM
9.6 9.2 8.8 8.4 8.0 7.6 7.2 6.8 6.4 6.0 5.6 5.2 4.8 4.4 4.0 3.6 3.2 2.8 2.4 2.0


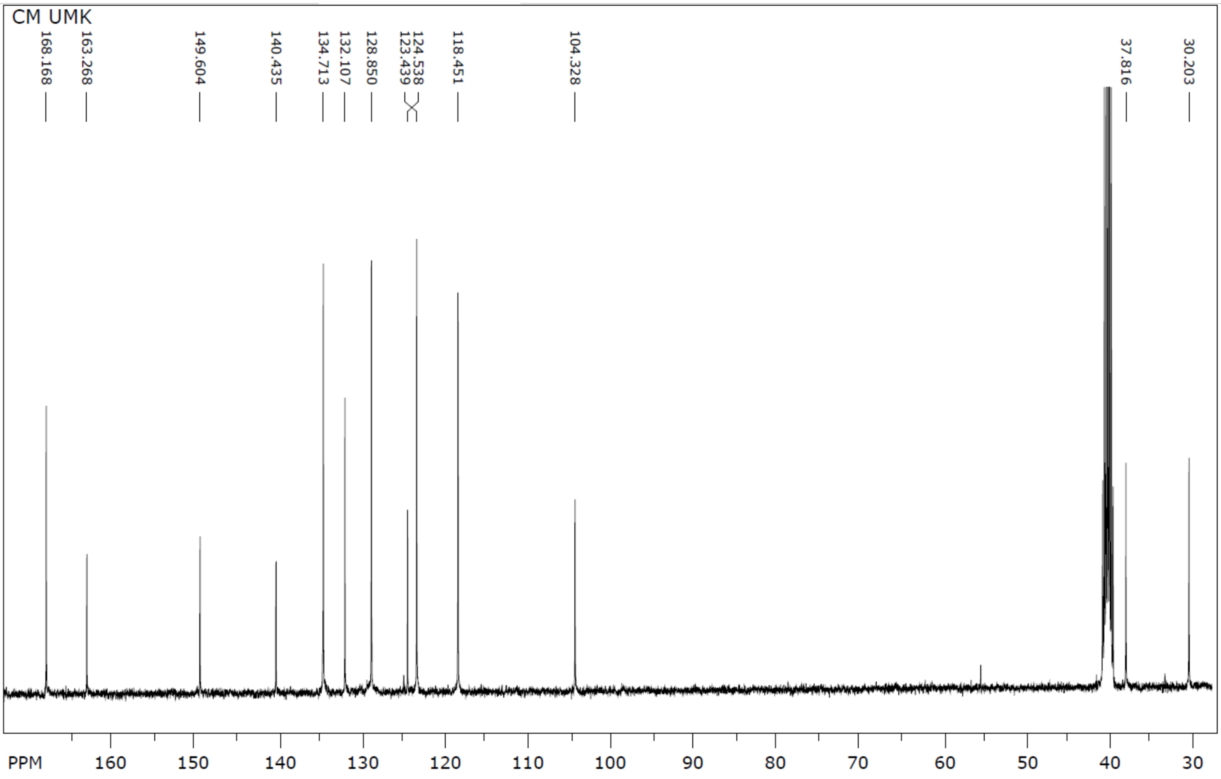

CM UMK
168.168
163.268
149.604
140.435
134.713
132.107
128.850
124.538
123.439
118.451
104.328
37.816
30.203
PPM
160 150 140 130 120 110 100 90 80 70 60 50 40 30

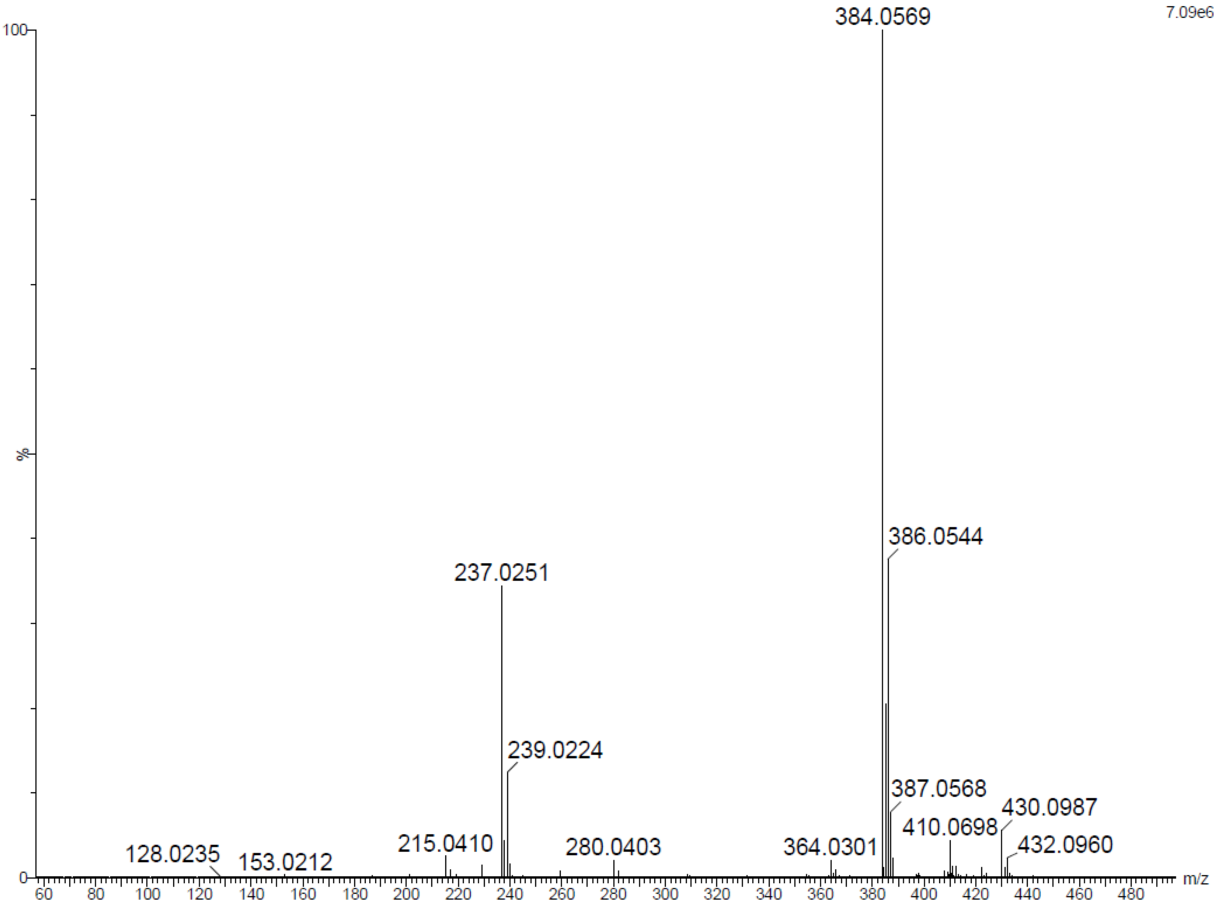
384.0569
7.09e6
100
%
0
386.0544
237.0251
239.0224
387.0568
430.0987
410.0698
432.0960
215.0410
280.0403
364.0301
128.0235
153.0212
60 80 100 120 140 160 180 200 220 240 260 280 300 320 340 360 380 400 420 440 460 480
m/z

Product **4c**

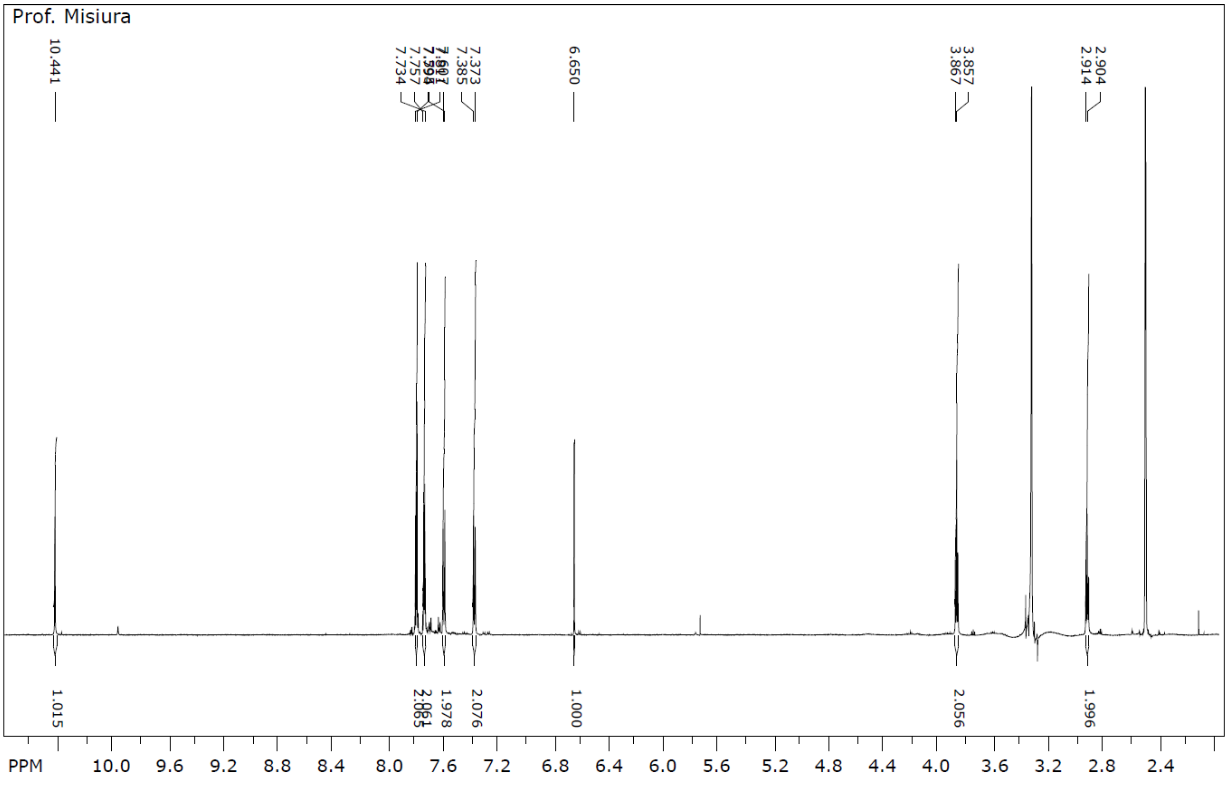
Prof. Misiura
10.441
7.734
7.757
7.373
7.385
6.650
3.857
3.867
2.904
2.914
1.015
1.978
2.076
1.000
2.056
1.996
PPM
10.0 9.6 9.2 8.8 8.4 8.0 7.6 7.2 6.8 6.4 6.0 5.6 5.2 4.8 4.4 4.0 3.6 3.2 2.8 2.4

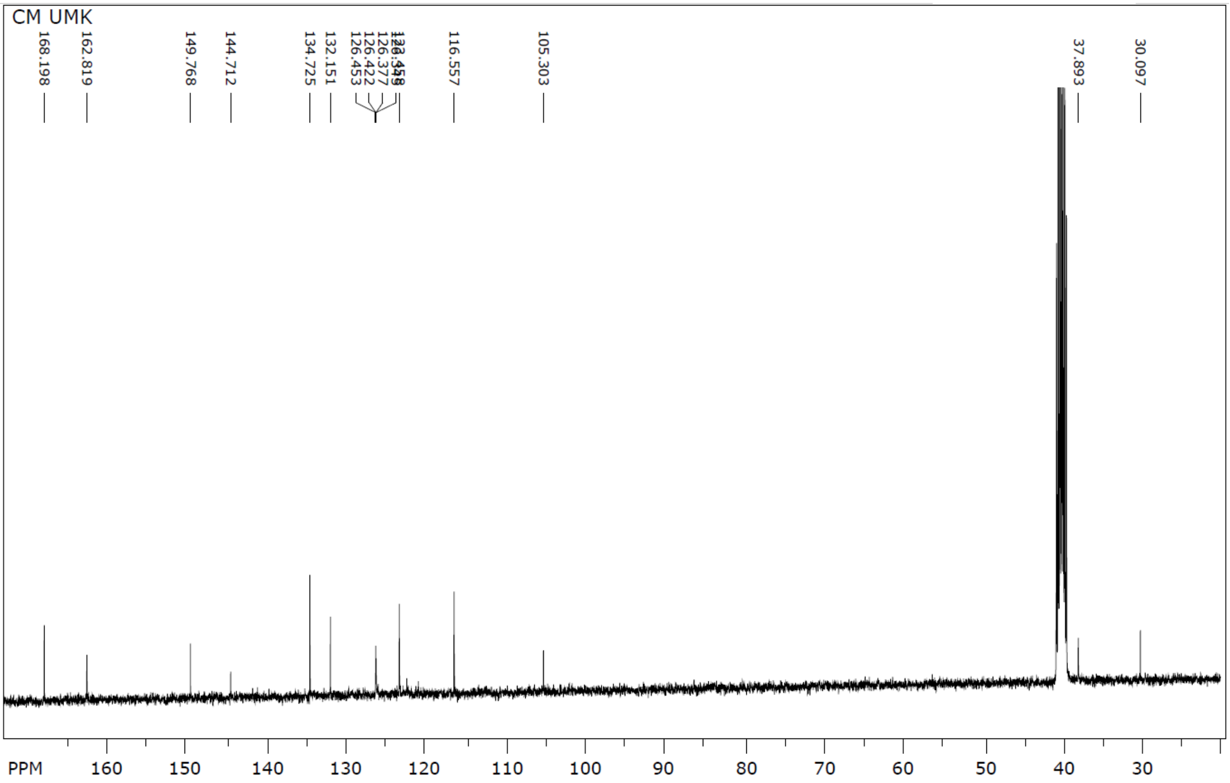
CM UMK
168.198
162.819
149.768
144.712
134.725
132.151
126.453
126.422
126.377
116.557
105.303
37.893
30.097
PPM
160
150
140
130
120
110
100
90
80
70
60
50
40
30

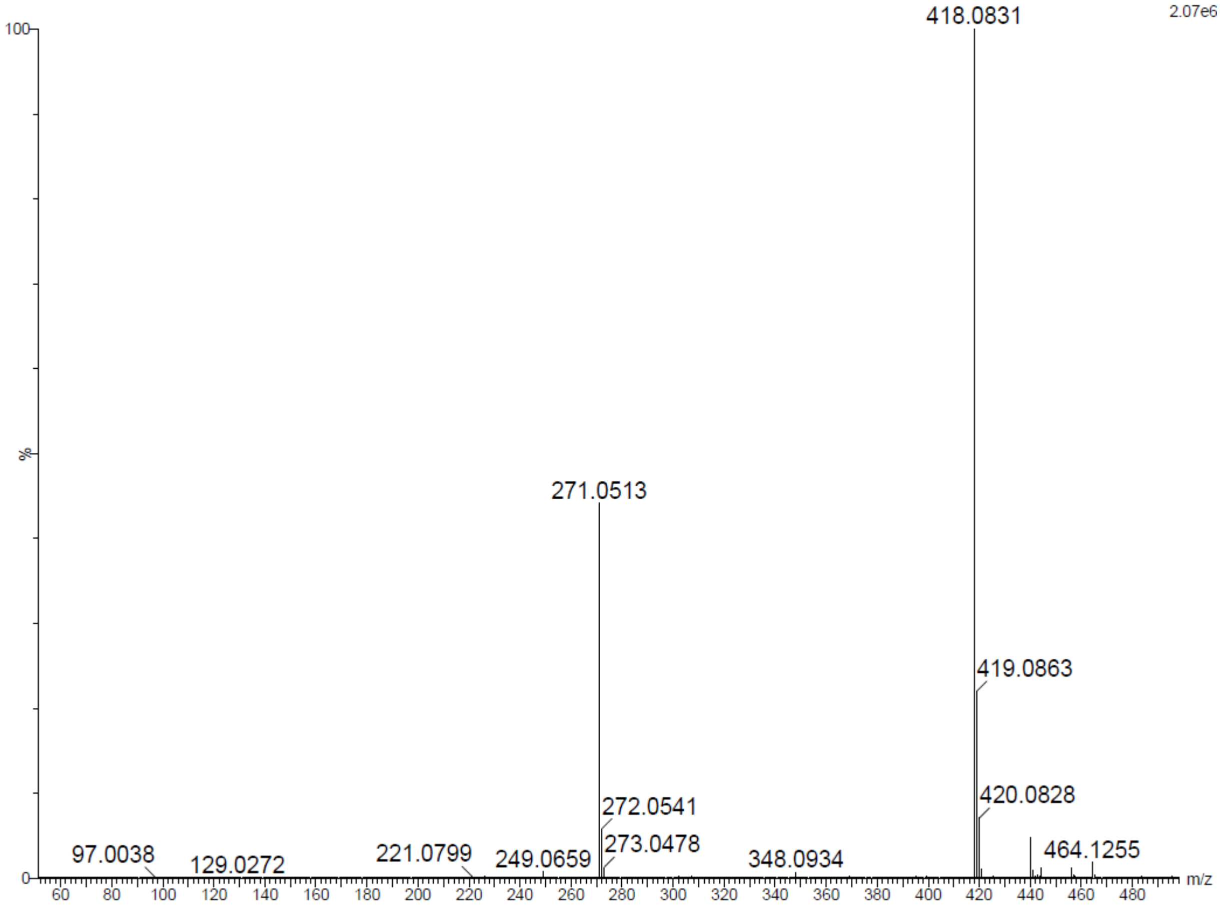
418.0831
2.07e6
100
%
0
271.0513
419.0863
420.0828
272.0541
273.0478
97.0038
129.0272
221.0799
249.0659
348.0934
464.1255
m/z
60
80
100
120
140
160
180
200
220
240
260
280
300
320
340
360
380
400
420
440
460
480

Product **4d**

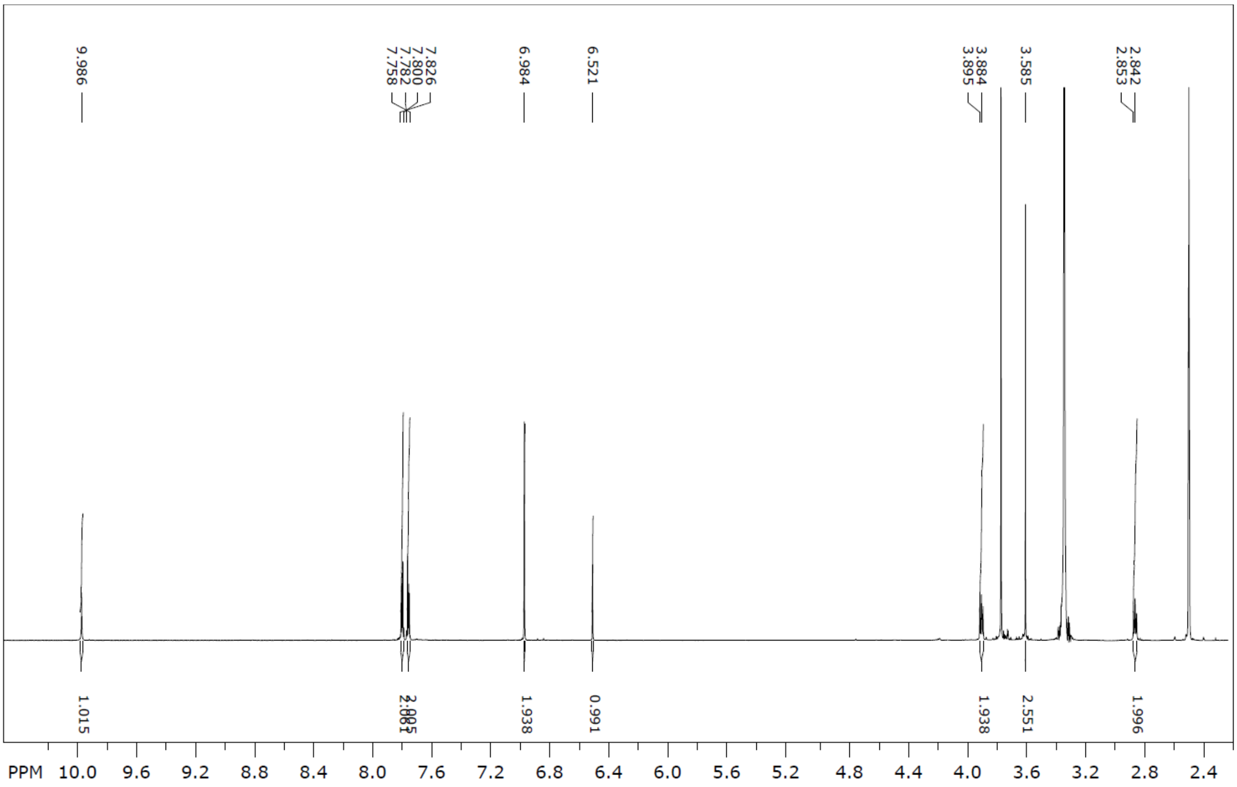

9.986
7.826
7.800
7.782
7.758
6.984
6.521
3.884
3.895
3.585
2.842
2.853
1.015
1.938
0.991
1.938
2.551
1.996
PPM 10.0 9.6 9.2 8.8 8.4 8.0 7.6 7.2 6.8 6.4 6.0 5.6 5.2 4.8 4.4 4.0 3.6 3.2 2.8 2.4


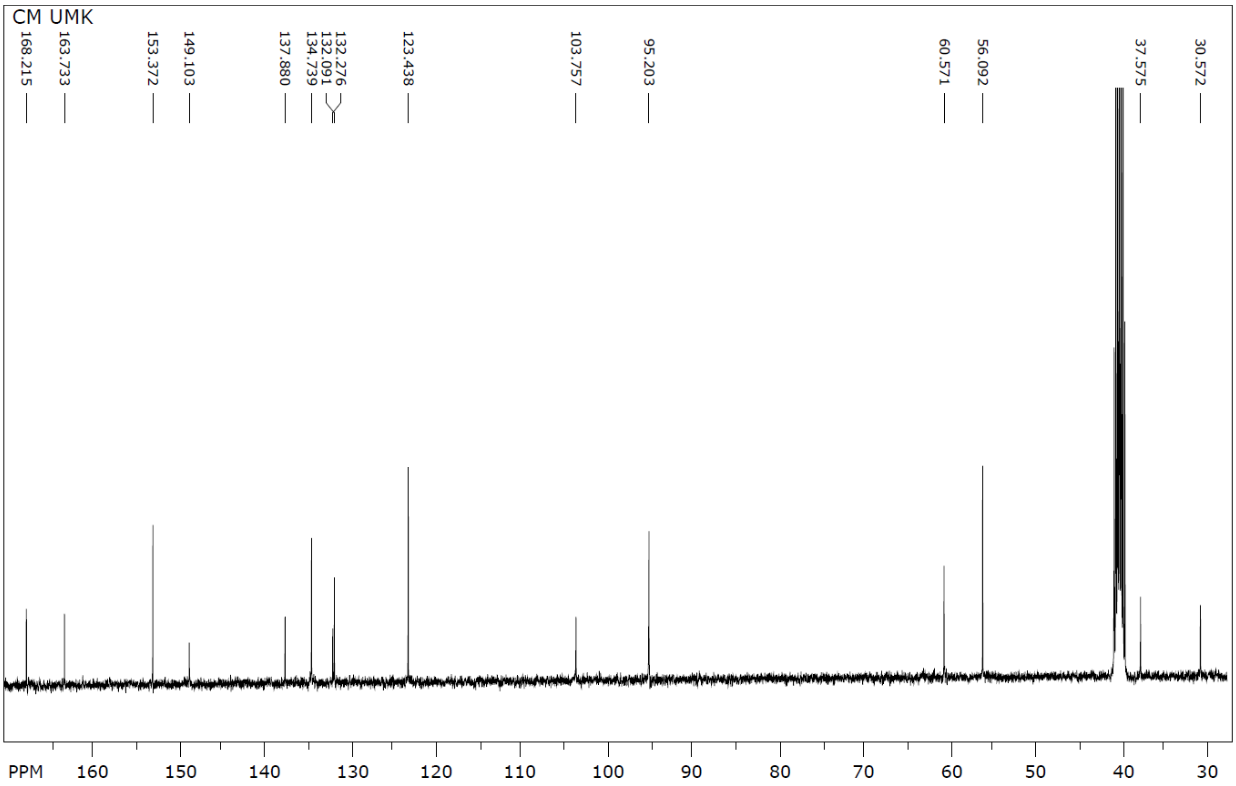

CM UMK
168.215
163.733
153.372
149.103
137.880
134.739
132.276
132.091
123.438
103.757
95.203
60.571
56.092
37.575
30.572
PPM 160 150 140 130 120 110 100 90 80 70 60 50 40 30

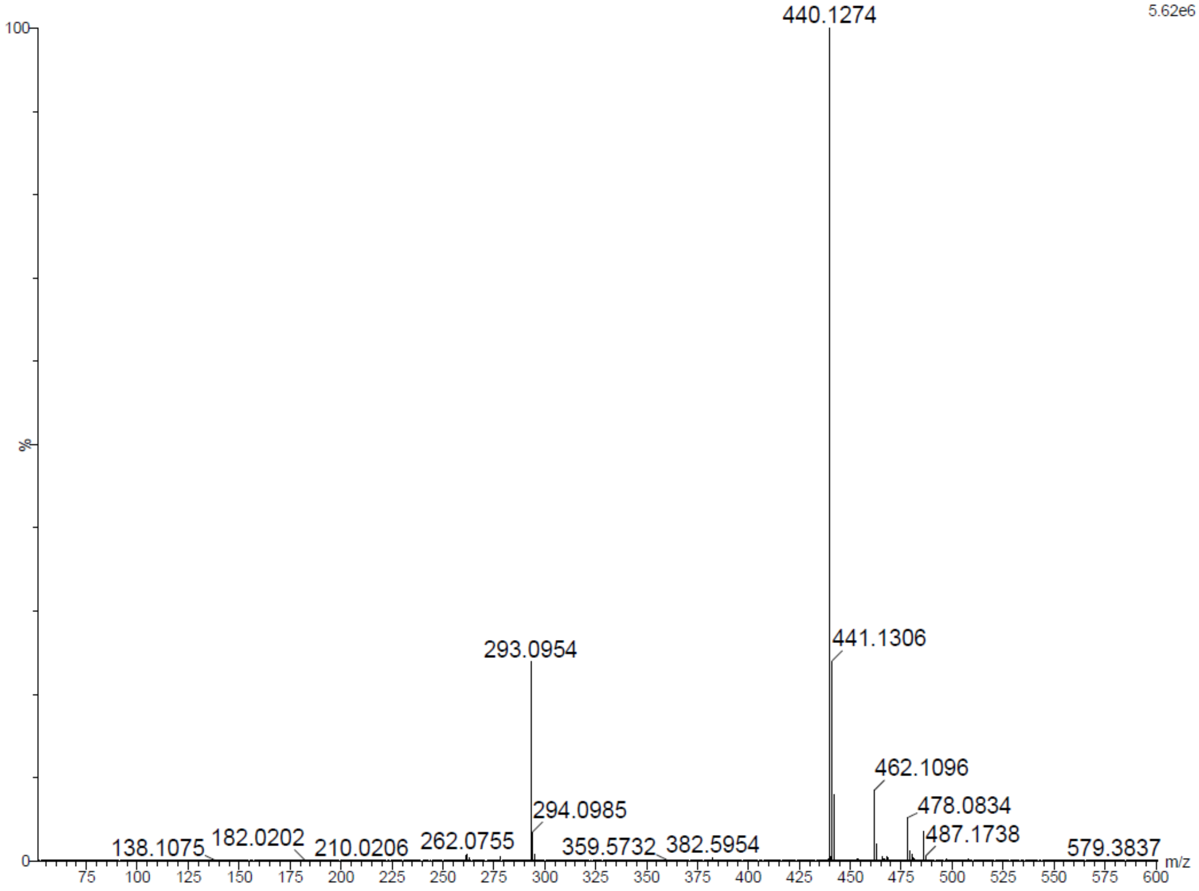

440.1274
5.62e6
100
%
0
293.0954
441.1306
462.1096
294.0985
478.0834
487.1738
138.1075
182.0202
210.0206
262.0755
359.5732
382.5954
579.3837
m/z
75 100 125 150 175 200 225 250 275 300 325 350 375 400 425 450 475 500 525 550 575 600


Product **4e**

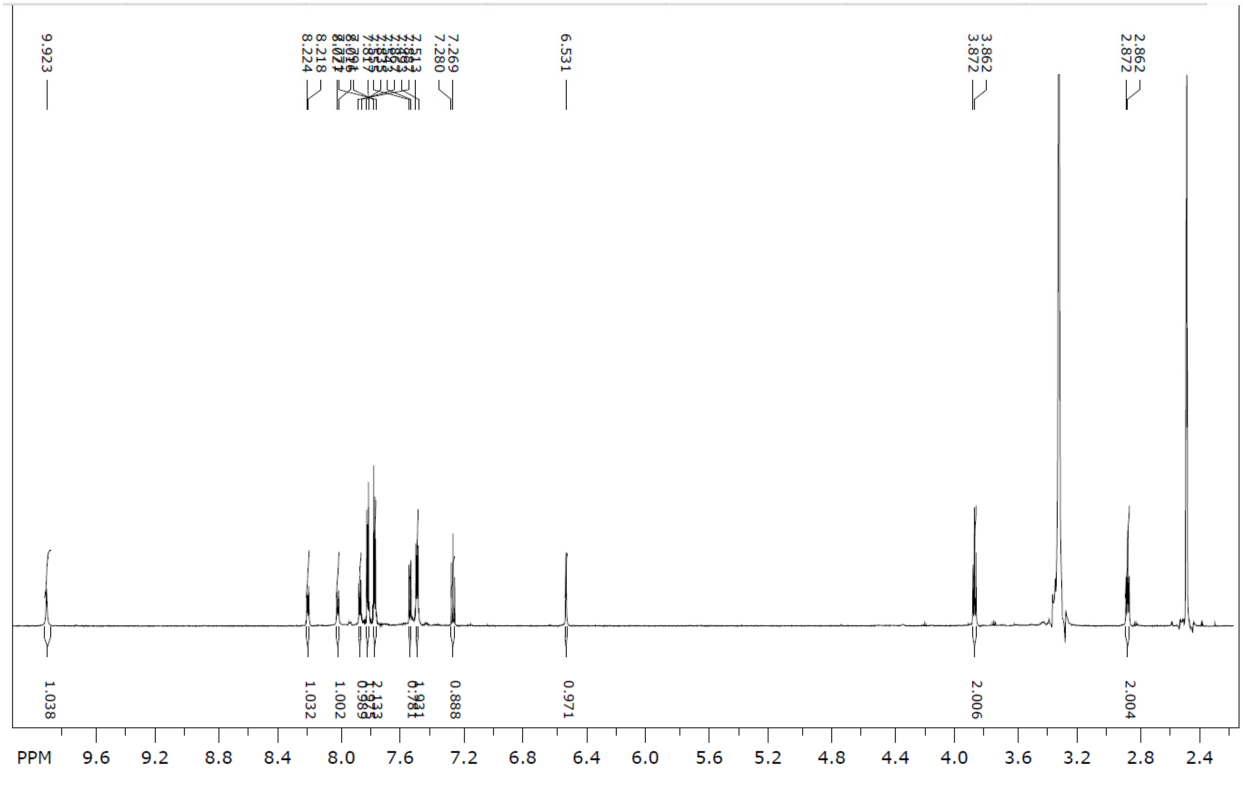

9.923
8.224
8.218
7.269
7.280
6.531
3.862
3.872
2.862
2.872
1.038
1.032
1.002
2.133
0.888
0.971
2.006
2.004
PPM 9.6 9.2 8.8 8.4 8.0 7.6 7.2 6.8 6.4 6.0 5.6 5.2 4.8 4.4 4.0 3.6 3.2 2.8 2.4

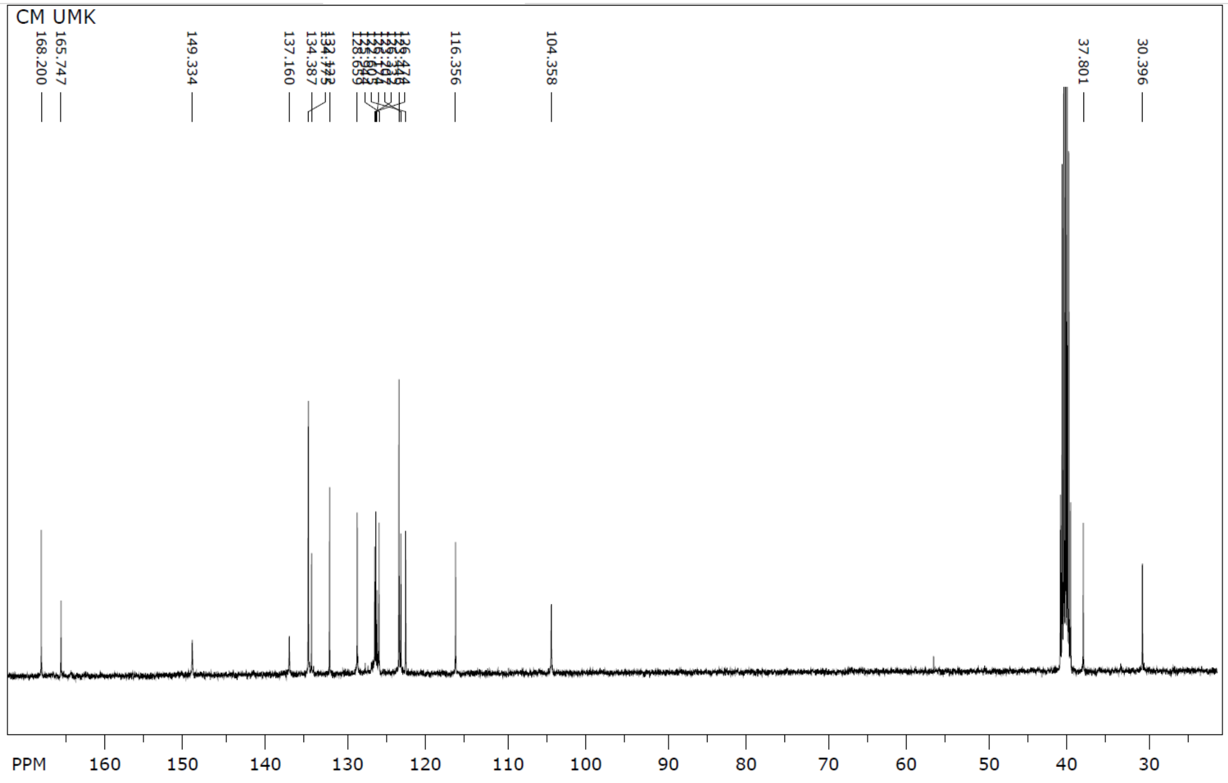

CM UMK
168.200
165.747
149.334
137.160
134.387
116.356
104.358
37.801
30.396
PPM
160
150
140
130
120
110
100
90
80
70
60
50
40
30


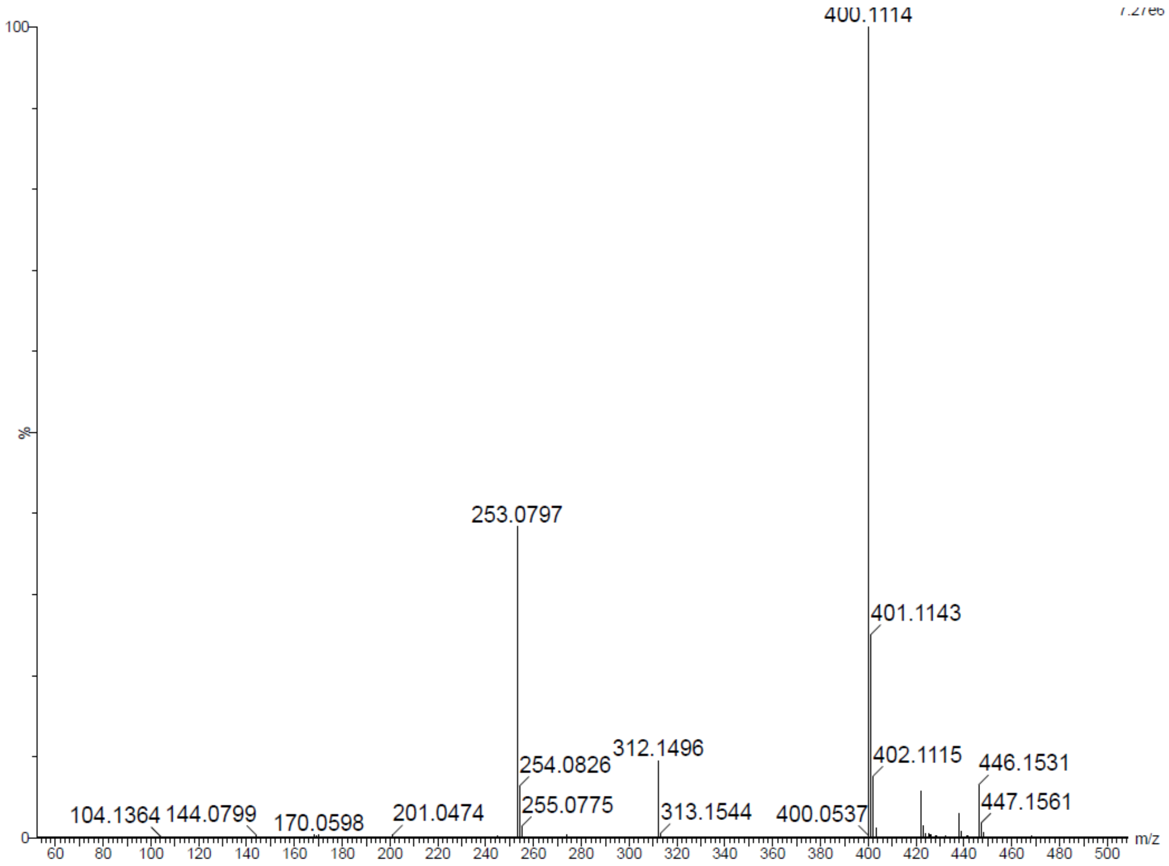

400.1114
100
%
253.0797
401.1143
312.1496
254.0826
402.1115
446.1531
447.1561
255.0775
104.1364
144.0799
170.0598
201.0474
313.1544
400.0537
m/z

Product **4f**

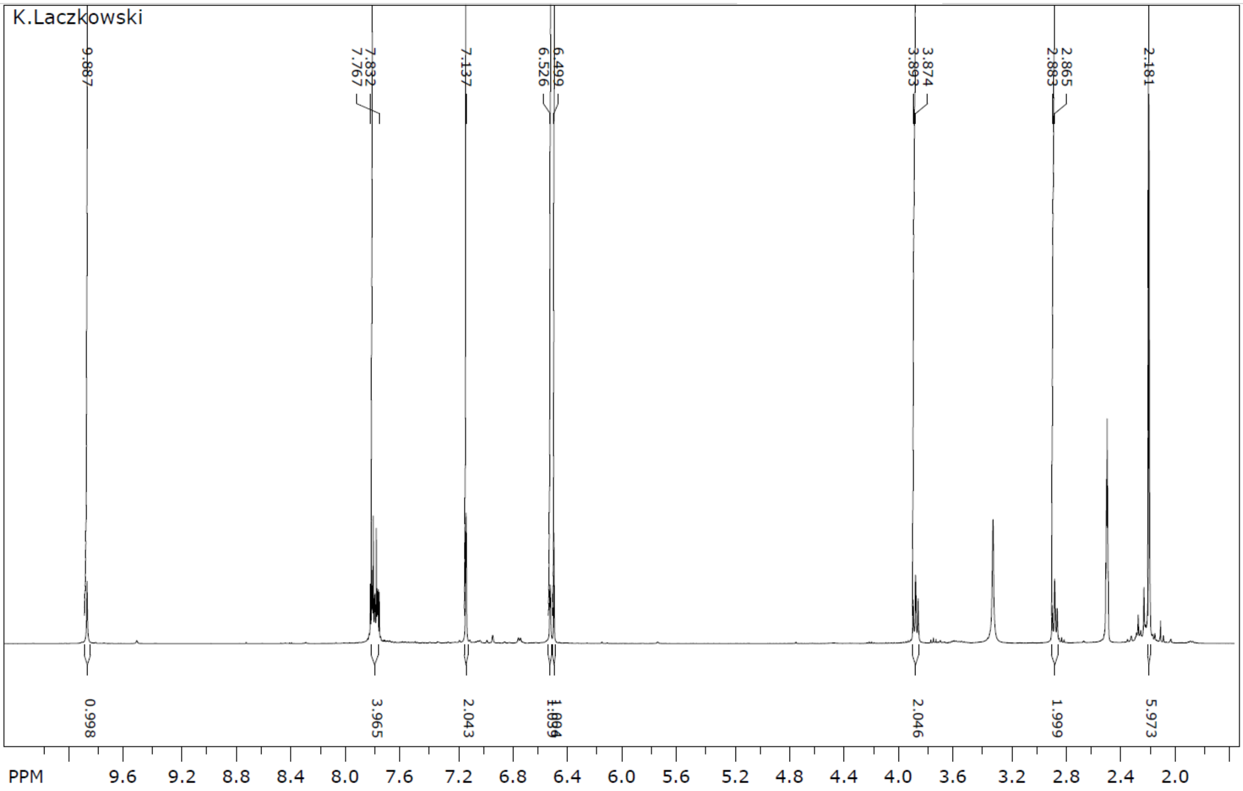
K.Laczkowski
9.887
7.832
7.767
7.137
6.526
6.499
3.874
3.893
2.865
2.883
2.181
0.998
3.965
2.043
2.046
1.999
5.973
PPM
9.6 9.2 8.8 8.4 8.0 7.6 7.2 6.8 6.4 6.0 5.6 5.2 4.8 4.4 4.0 3.6 3.2 2.8 2.4 2.0

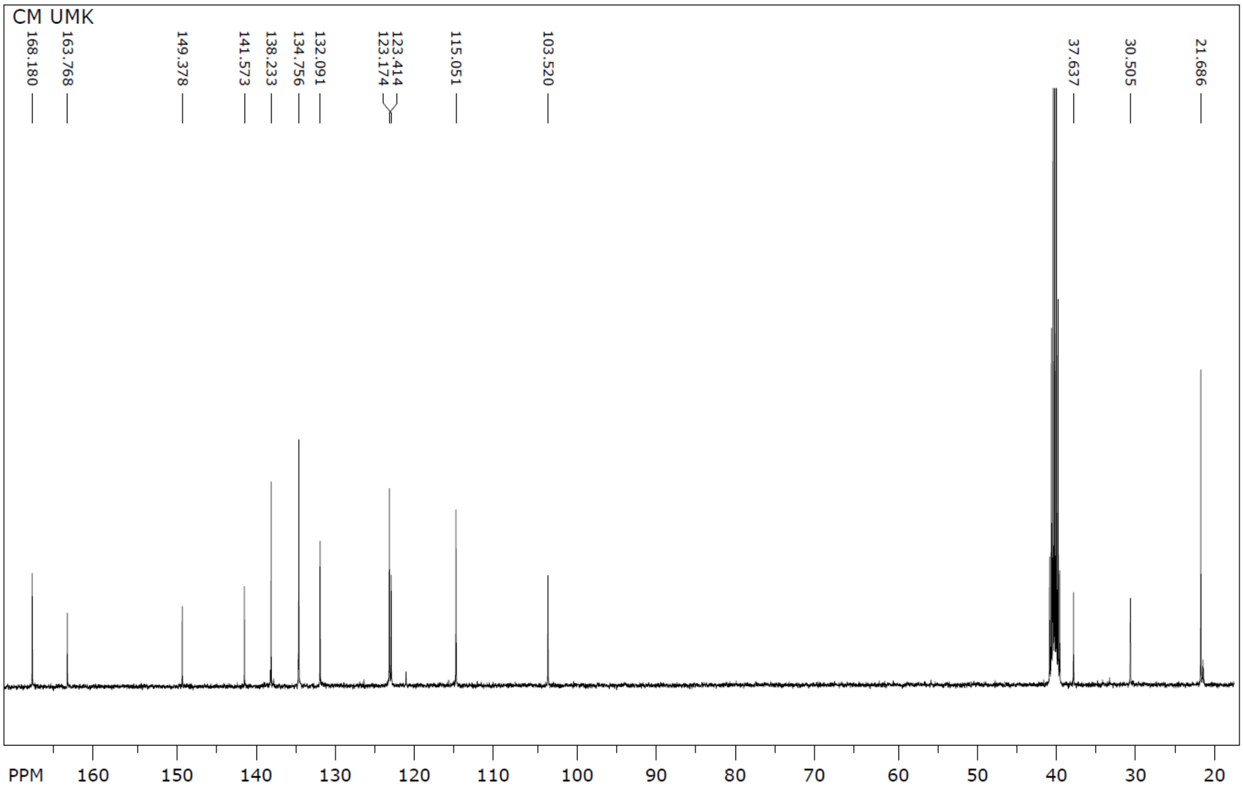
CM UMK
168.180
163.768
149.378
141.573
138.233
134.756
132.091
123.414
123.174
115.051
103.520
37.637
30.505
21.686
PPM
160 150 140 130 120 110 100 90 80 70 60 50 40 30 20

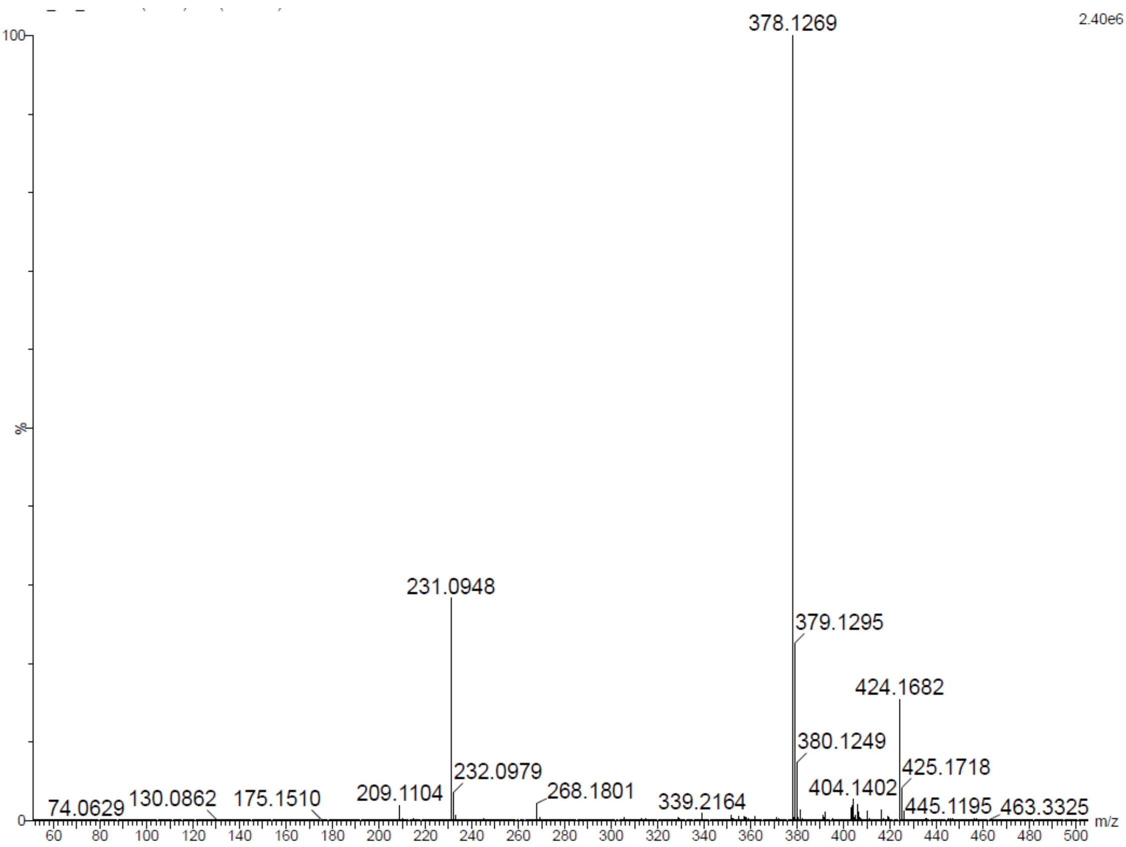
378.1269
2.40e6
100
%
0
231.0948
379.1295
424.1682
380.1249
232.0979
425.1718
74.0629
130.0862
175.1510
209.1104
268.1801
339.2164
404.1402
445.1195
463.3325
m/z

Product **4g**

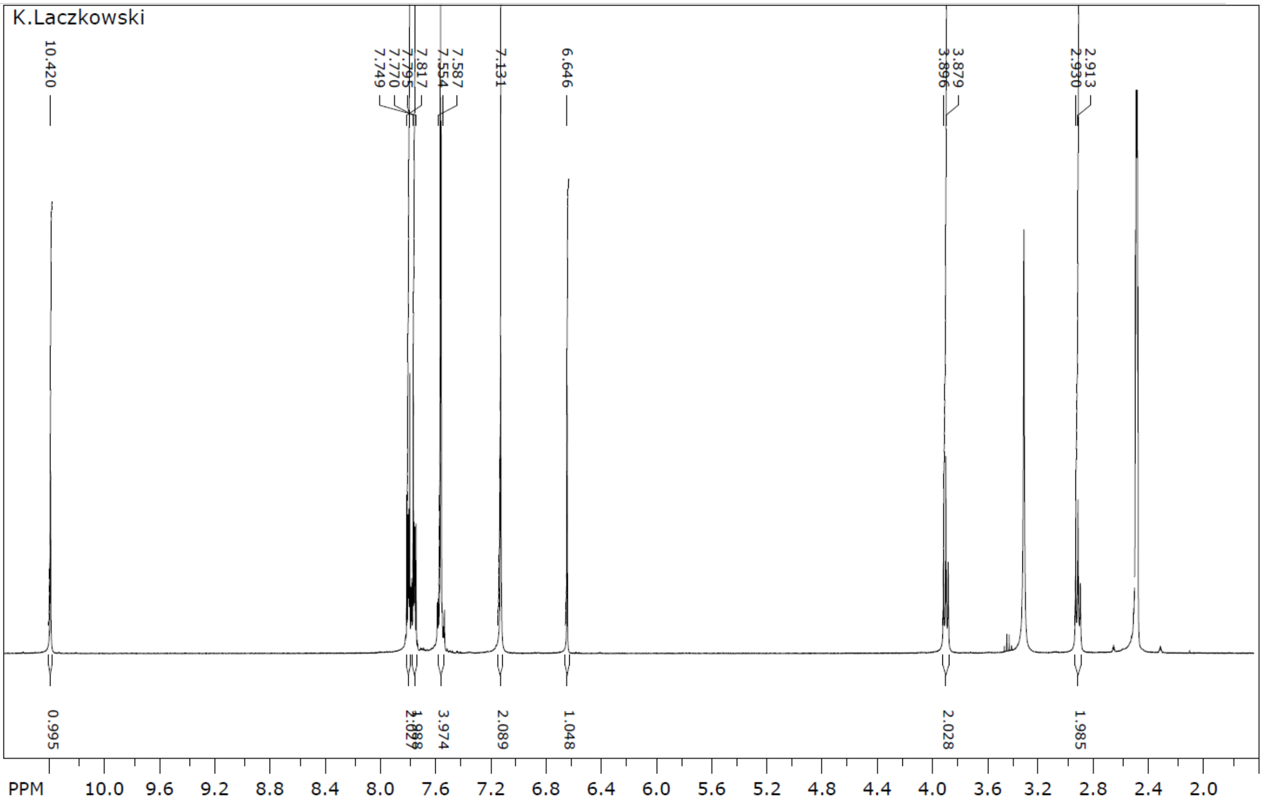
K.Laczkowski
10.420
7.817
7.795
7.770
7.749
7.587
7.554
7.131
6.646
3.896
3.879
2.930
2.913
0.995
3.974
2.089
1.048
2.028
1.985
PPM

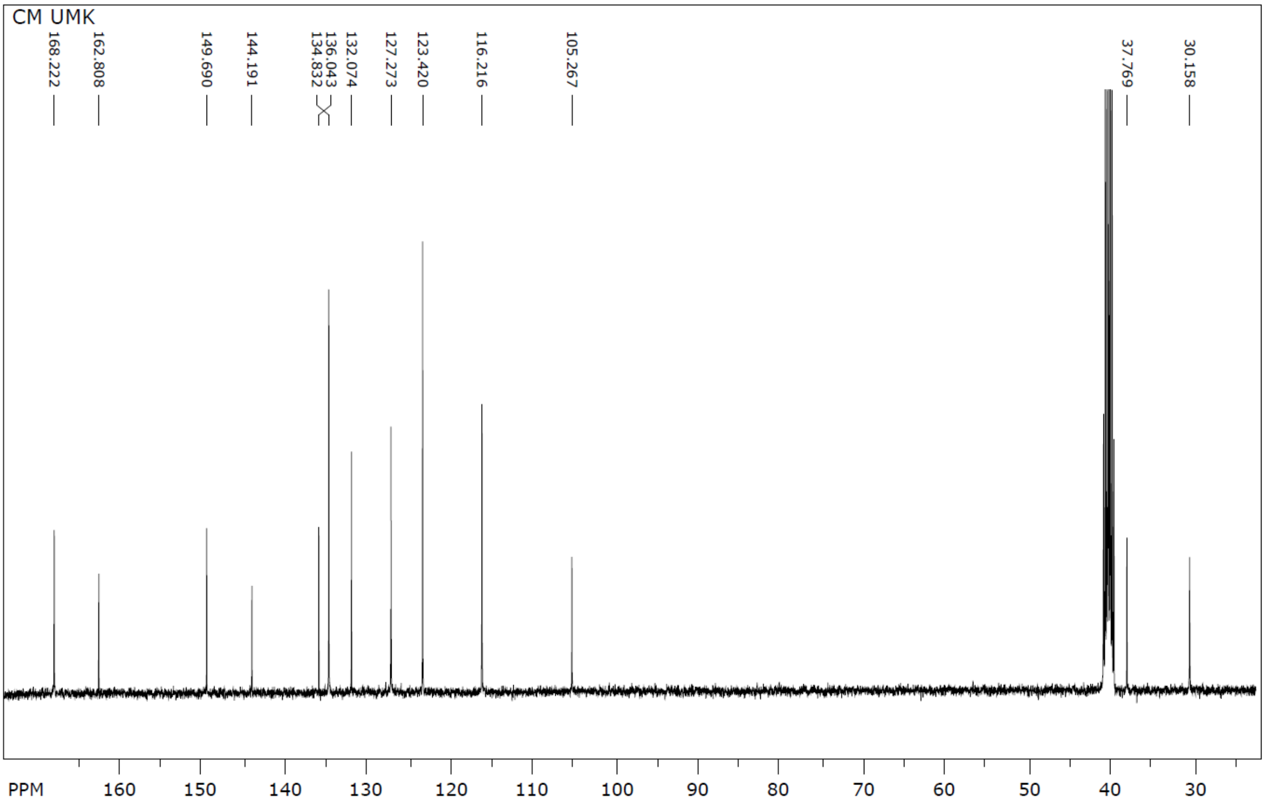
CM UMK
168.222
162.808
149.690
144.191
136.043
134.832
132.074
127.273
123.420
116.216
105.267
37.769
30.158
PPM
160 150 140 130 120 110 100 90 80 70 60 50 40 30

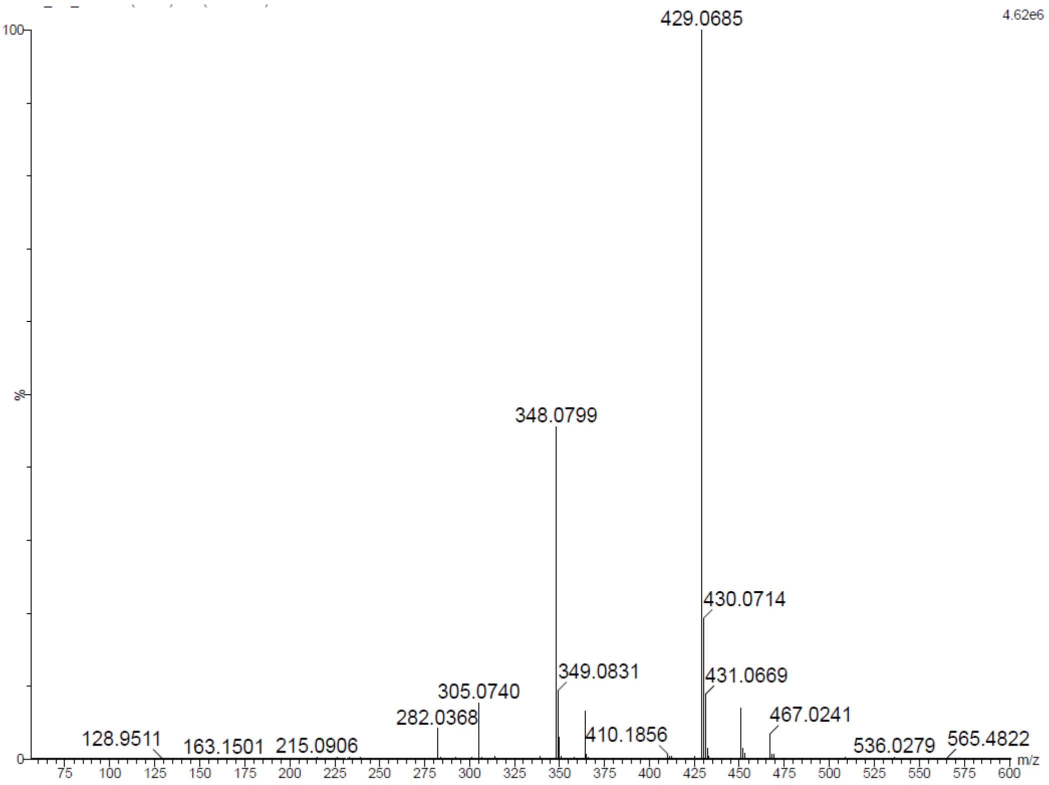
4.62e6
100
%
0
429.0685
348.0799
430.0714
349.0831
431.0669
305.0740
282.0368
467.0241
410.1856
128.9511
163.1501
215.0906
536.0279
565.4822
75 100 125 150 175 200 225 250 275 300 325 350 375 400 425 450 475 500 525 550 575 600
m/z

Product **4h**

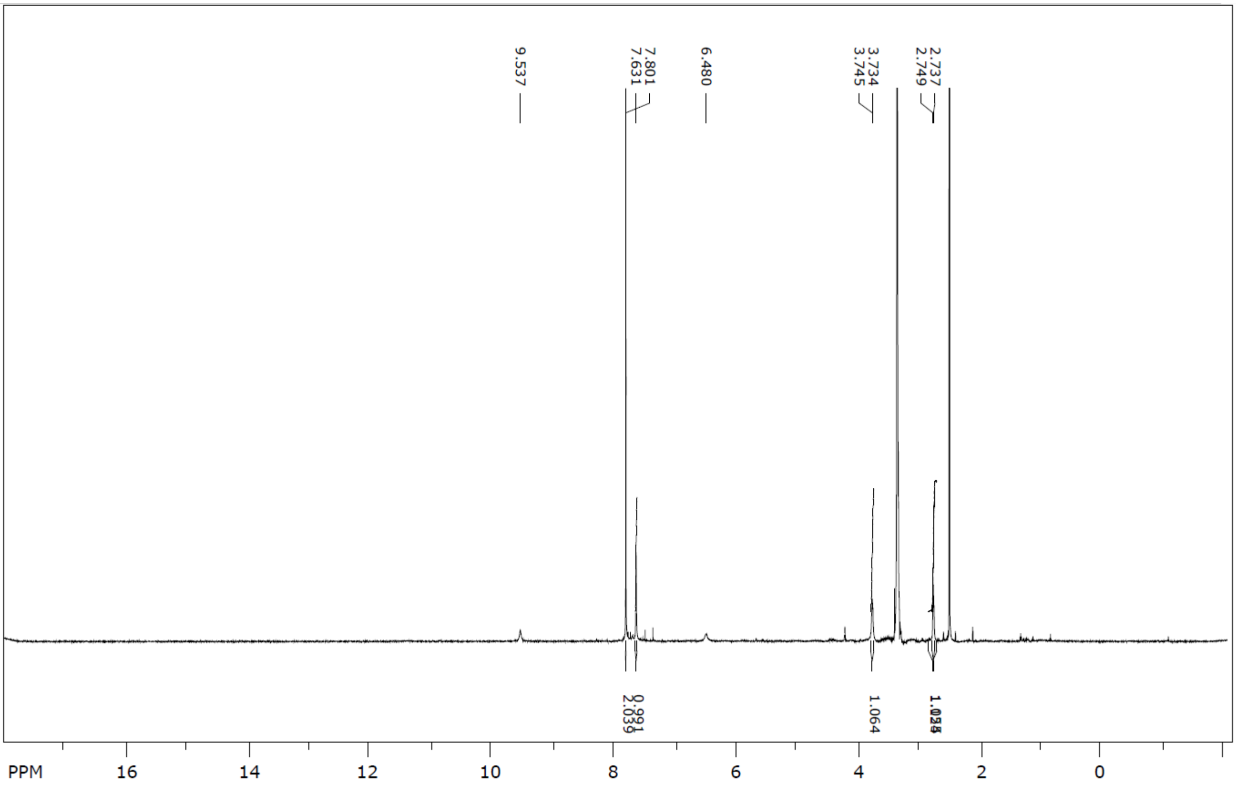
9.537
7.801
7.631
6.480
3.734
3.745
2.737
2.749
2.039
0.991
1.064
PPM
16
14
12
10
8
6
4
2
0

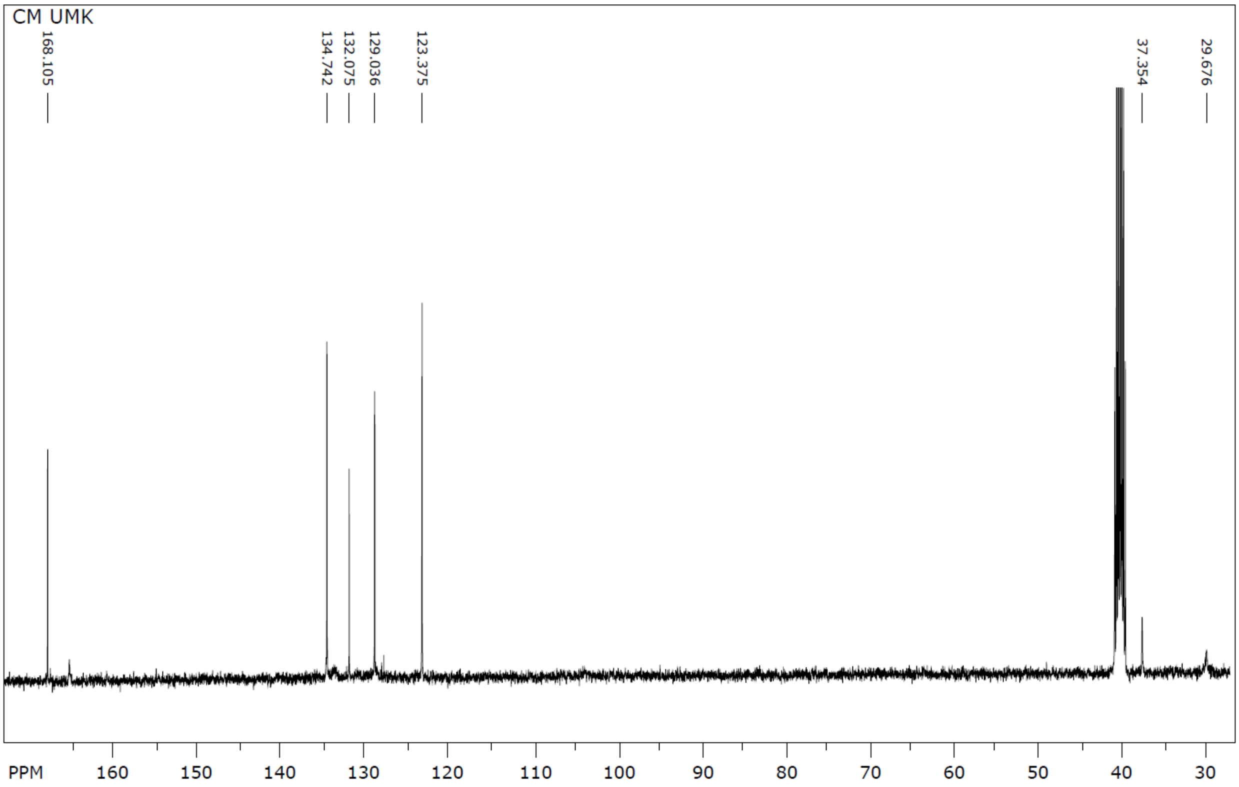
CM UMK
168.105
134.742
132.075
129.036
123.375
37.354
29.676
PPM
160
150
140
130
120
110
100
90
80
70
60
50
40
30

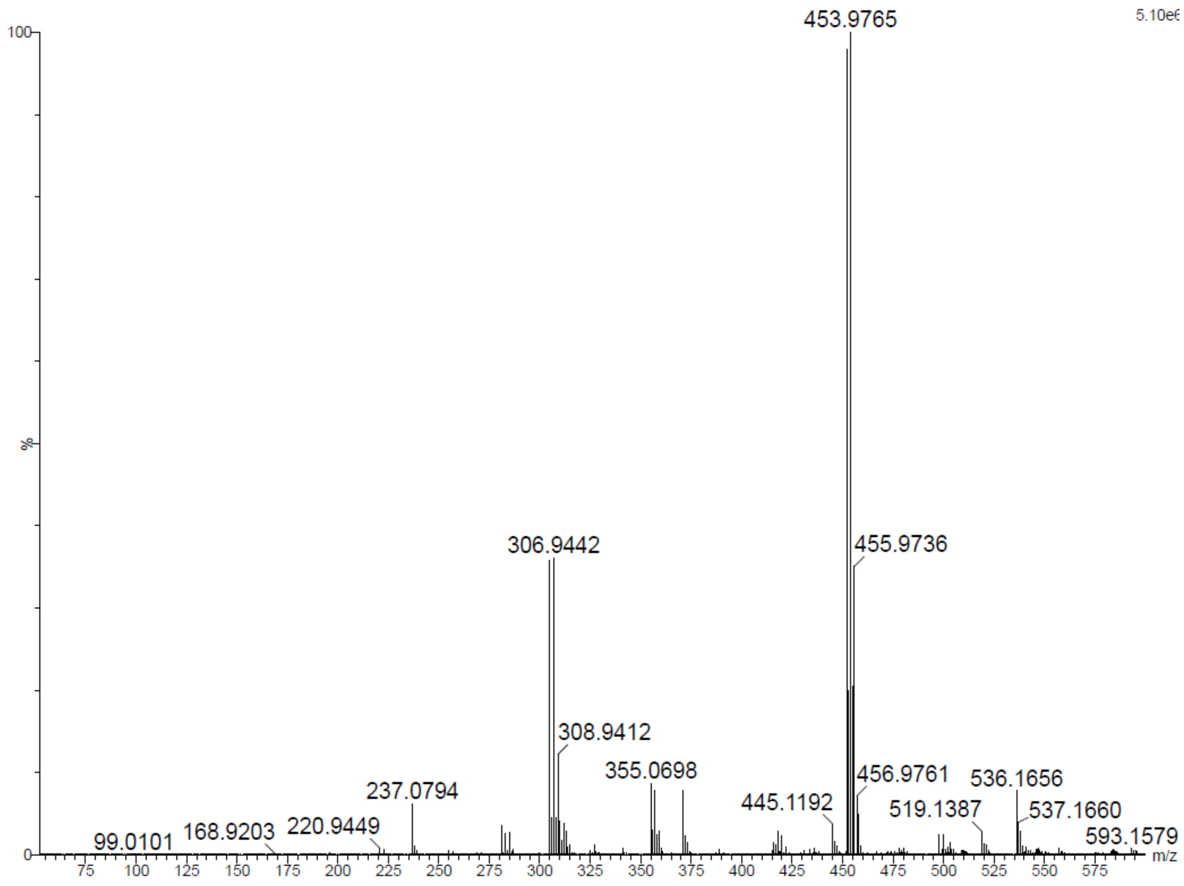

453.9765
5.10e6
100
%
0
306.9442
455.9736
308.9412
355.0698
456.9761
536.1656
237.0794
445.1192
519.1387
537.1660
99.0101
168.9203
220.9449
593.1579
m/z


Product **4i**

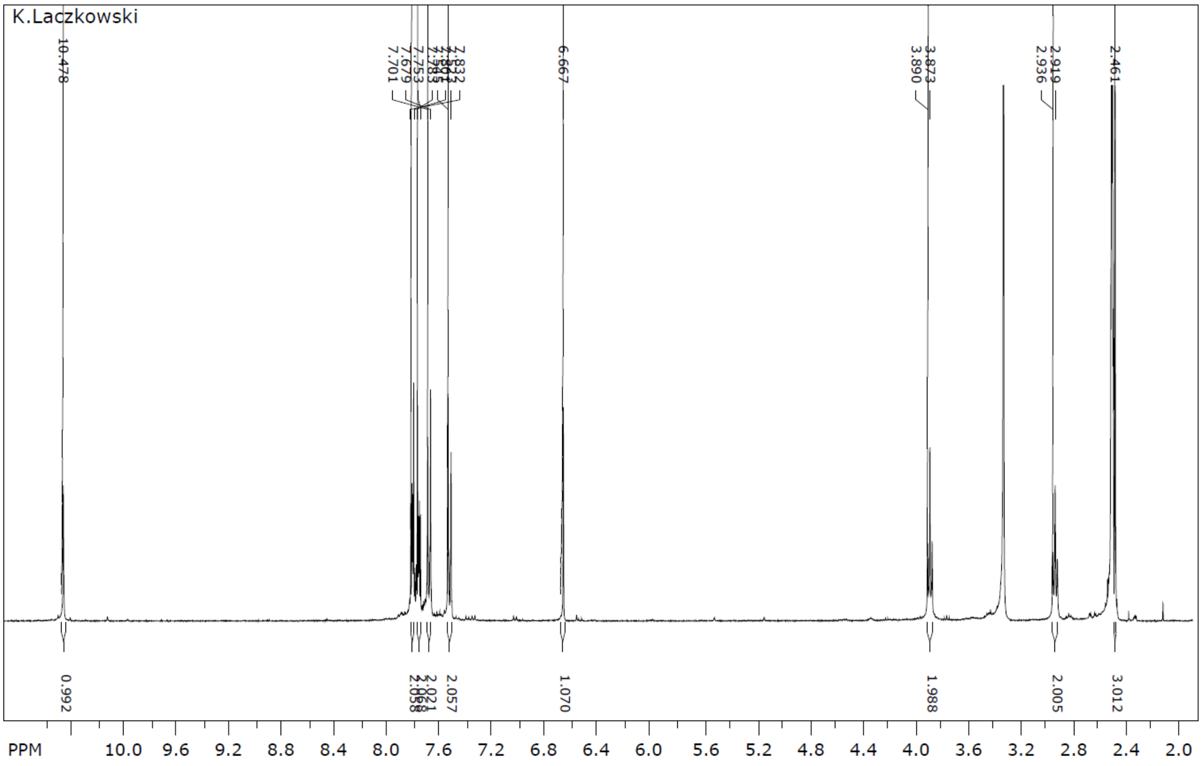

K.Laczkowski
10.478
7.832
7.701
7.679
7.753
6.667
3.890
3.873
2.936
2.919
2.461
0.992
2.057
1.070
1.988
2.005
3.012
PPM

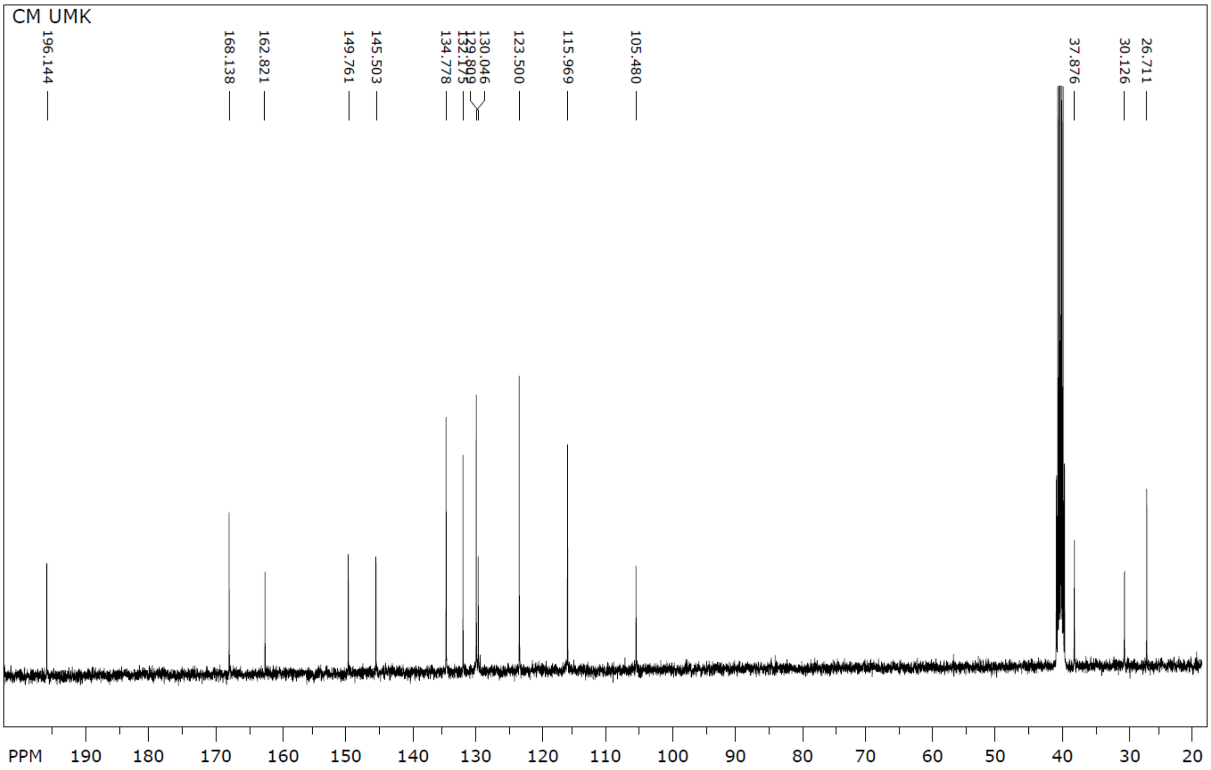
CM UMK
196.144
168.138
162.821
149.761
145.503
134.778
132.175
129.809
130.046
123.500
115.969
105.480
37.876
30.126
26.711
PPM

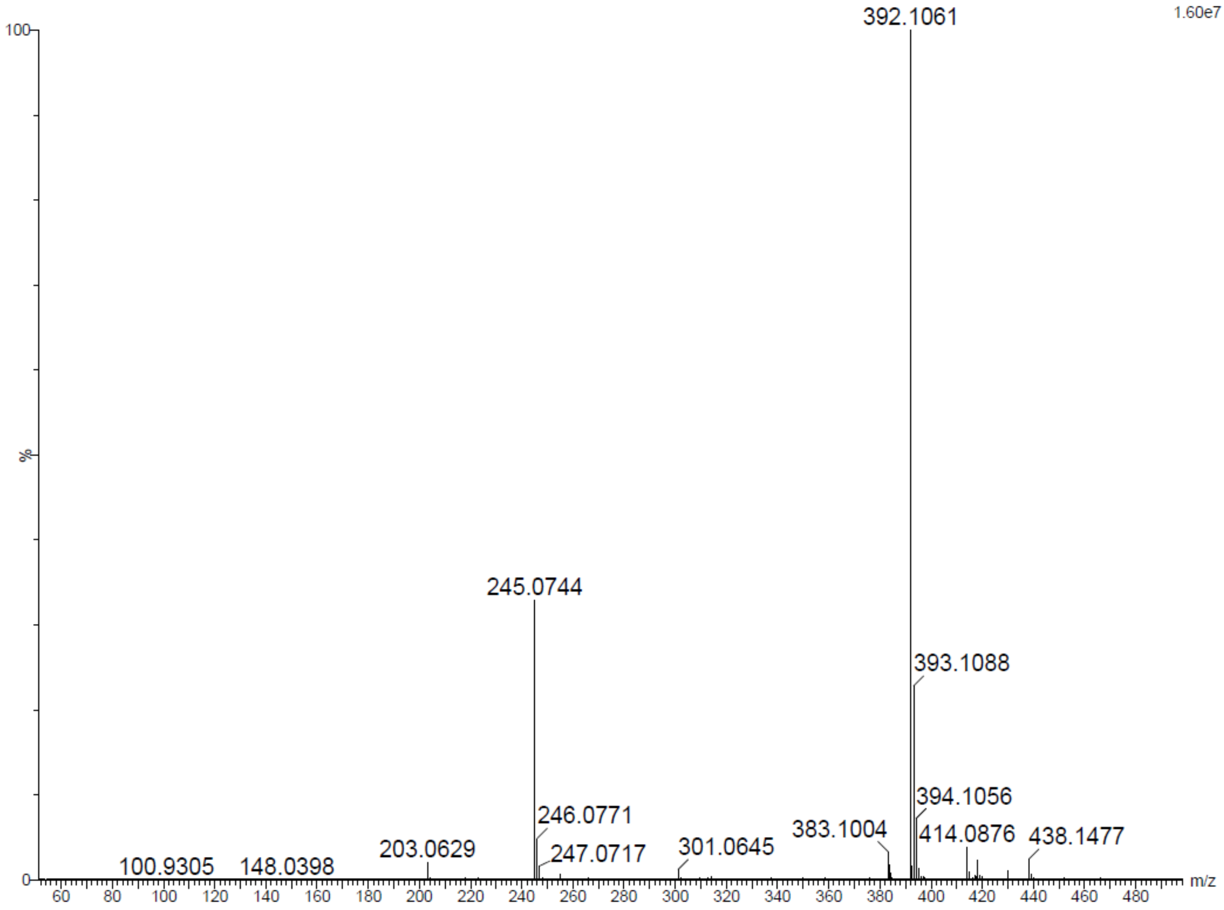
392.1061
1.60e7
245.0744
393.1088
394.1056
246.0771
414.0876
438.1477
383.1004
301.0645
247.0717
203.0629
100.9305
148.0398
m/z
%
